# Vector-stimuli-responsive magnetorheological fibrous materials

**DOI:** 10.1038/s41586-025-09706-4

**Published:** 2025-11-05

**Authors:** Junhong Pu, Haiqiong Li, Jin Liu, Ke Li, Xiaoming Tao

**Affiliations:** 1 Research Institute for Intelligent Wearable Systems, The Hong Kong Polytechnic University, Hong Kong, China; 2 School of Fashion and Textiles, The Hong Kong Polytechnic University, Hong Kong, China; 3 PolyU-Jinjiang Technology and Innovation Research Institute, Jinjiang, China

**Keywords:** Polymers, Actuators, Composites

## Abstract

Fibrous materials that provide reversible actuation^1,2^ or adapt mechanical properties^3,4^ in response to external stimuli hold great promise for smart textiles^5^, soft robotics^6^ and wearable technologies^7^. Although considerable progress has been made in creating fibrous materials responsive to scalar stimuli such as voltage^8^, temperature^6^, humidity^2^ and ion concentration^9^, these technologies often lack directional controllability and functional diversity^10–14^. Here we report a class of vector-stimuli-responsive magnetorheological fibrous materials, guided by our engineering model integrating the structural mechanics of textiles with the magnetics of soft magnetic materials. We mass-produced soft magnetic polymer composite fibres with optimized mechanical and magnetic properties, which we then assembled into concentric helical yarns. These yarns exhibited pronounced bending and stiffening properties controlled by the direction and magnitude of magnetic fields, allowing for customized fabrics with various actuation and stiffening functionalities. We demonstrated innovative smart textiles derived from those fabrics, including an active ventilation fabric for personal moisture management, an integrated conformable gripper for handling objects of varying shapes and stiffness, and a compact remote-controllable haptic finger glove that replicates the sensation of fabric hardness and smoothness. Our work provides insights into stimuli-responsive fibrous materials, elevating them from scalar to sophisticated vector control, heralding an era of smart textile innovation.

## Abstract (web summary)

Vector-stimuli-responsive magnetorheological fibrous materials can be mass-produced, which can be assembled into yarns and woven into a variety of fabrics with actuation and stiffening abilities.

## Main

Magnetorheological (MR) materials, a class of smart materials that can reversibly change rheological and mechanical properties under magnetic fields^15–17^, are composed of soft magnetic particles within a fluid or elastomeric carrier^18,19^. Under external magnetic fields, the magnetized particles attract each other through dipole–dipole interaction to form fibre-like structures—known as the MR effect—that increase the viscosity and stiffness of the MR materials^20,21^. Among these materials, anisotropic MR elastomers with predefined fibre-like soft magnetic structures exhibit directional responses, including sheer stiffening^22^ and rotational actuation^23^, to magnetic fields. However, their performance levels are limited because of the inherent rigidity of elastomer matrices^22,24^, necessitating high magnetic field strengths that are unsafe for human proximity^25,26^. Recently, magnetic fibres containing hard-magnetic particles have shown marked potential in soft robotics, medical devices and textile-based bioelectronics^27–31^. However, their use as textile actuators presents several challenges. Integrating magnetized fibres into fabrics with predefined magnetization patterns is limited by inter-fibre magnetic interaction^32,33^, relatively low precision in textile manufacturing, and the inherently non-bonded hierarchical fabric structures, collectively causing dislocated or disordered magnetization patterns. Magnetizing the fabrics directly is limited because of magnetizer spatial constraints, typically restricting the fabric size to a few centimetres (ref. ^34^). Moreover, magnetic interactions between magnetized parts during actuation hinder stable, controllable and reversible movements in fabrics, which allow for hierarchical internal relative motion^23^.

**Fig. 1:**
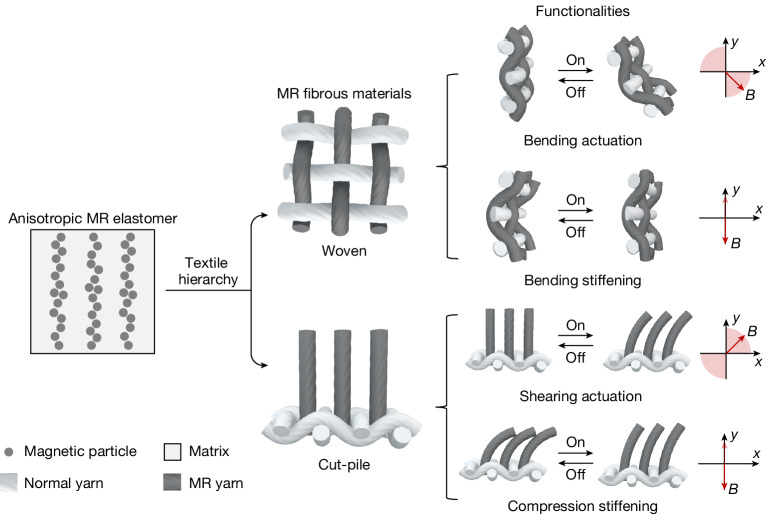
Schematic of MR fibrous materials with vector-stimuli-responsive functionalities. Central to the design of anisotropic MR elastomers is their fibre-like architecture, densely packed with magnetic particles embedded in an elastomeric matrix. Although this inactive matrix is essential for structural integrity, it diminishes the performance of the magnetic-active structure. Inspired by this magnetically anisotropic architecture, MR fibrous materials, exemplified by woven and cut-pile MR fabrics, have been innovated. These MR fabrics, characterized by their intrinsic magnetic anisotropy, eliminate the inactive matrix by the textile assembly of MR yarns, thereby enabling fibrous materials with vector-stimuli-responsive behaviour. This advancement results in MR fabrics that offer a range of sophisticated functionalities not achievable with conventional scalar-stimuli-responsive fibrous materials. For example, a woven MR fabric, when fixed at one end, bends in response to non-parallel magnetic fields. By contrast, it stiffens under a vertical magnetic field, exhibiting reduced bending deformation under the same bending moment. Similarly, the cut-pile MR fabric demonstrates surface shearing under non-parallel magnetic fields, whereas under a vertical magnetic field, it significantly stiffens to resist compression. The range of magnetic field directions is indicated in the related Cartesian coordinate systems.

We proposed that MR fibrous materials could synergize the vector-stimuli-responsive behaviour of anisotropic MR materials with the flexibility and versatility of textiles, yielding a transformative class of vector-stimuli-responsive fibrous materials. Moreover, soft magnetic MR fibres do not have the limitations of hard-magnetic fibres in textile actuators, as they require no pre-magnetization. These magnetically anisotropic and matrix-free MR fibrous materials, enabled by non-bonding fibre assembly, can achieve high-performance actuation and stiffening functionalities previously unattainable (Fig. 1). Furthermore, the extensibility and programmability of textiles facilitate seamless transitions from two-dimensional fabrics to complex three-dimensional (3D) textile devices for practical applications.

Two fundamental challenges hinder the development of high-performance magnetic fibrous materials, including fibres, yarns and fabrics. First, fibre spinning techniques face a contradiction between high filler content and thin fibre diameter^35,36^, compromising functional and mechanical properties. Second, the understanding of the multilevel structure–property relationship of stimuli-responsive fibrous materials is still lacking^5,7^, leading to notable performance decay and inadequate functionalities on the hierarchical assembly of textile structures. Solution spinning techniques such as wet spinning and electrospinning yield fibres with diameters less than 100 μm, but typically containing filler content under 20 wt% (refs. ^37–39^). Extrusion-based fibre processes, such as melt spinning and printing, produce fibres with filler content above 40 wt%. However, high loading can cause filler aggregation and undesirable rheological properties, leading to instability during extrusion and drawing, which results in fibre diameters often exceeding 200 μm (refs. ^40,41^). This restricts the concurrent achievement of high magnetic susceptibility and flexibility of MR fibres. For fibre assemblies, non-woven fabrics stacked by electrospun fibres exhibit limited durability and processability^39^, whereas woven and knitted fabrics integrated by monofilaments often show suboptimal flexibility and deficient resilience^34,41^.

## Design of MR fibres and yarns

**Fig. 2:**
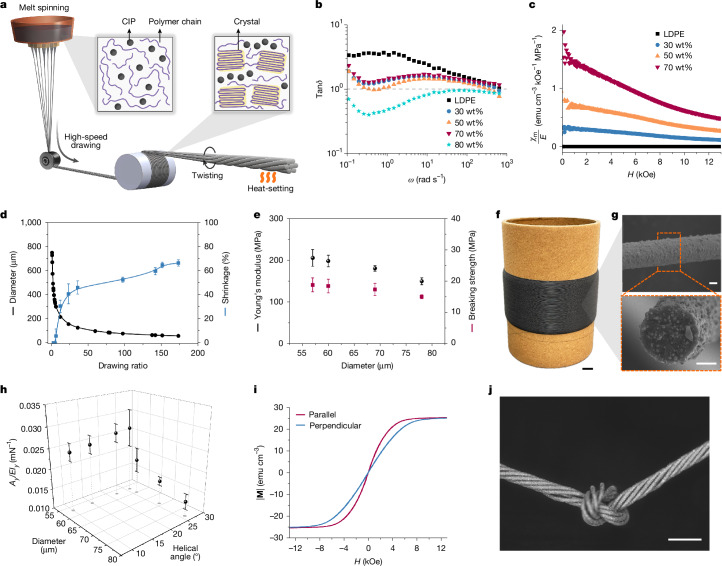
Design and fabrication of MR fibres and yarns. **a**, Schematic of the melt spinning process with in situ high-speed drawing, showcasing the uniform dispersion of CIPs and the random alignment of polymer chains in the melt, which become lengthwise oriented in the MR fibres. These fibres are subsequently twisted into MR yarns followed by heat-setting. **b**, Logarithmic plots of loss factor as a function of angular frequency of molten LDPE and composites. **c**, *χ*_m_/*E*, serving as a criterion for identifying soft magnetic materials with high magnetic activation ability, of LDPE and composites across the magnetic field spectrum. **d**, Diameter and axial thermal shrinkage of MR fibres with different drawing ratios. Error bars correspond to standard deviations (s.d.) (*n* = 5). **e**, Young’s modulus and breaking strength of MR fibres with different diameters. Error bars correspond to s.d. (*n* = 5). **f**, Photograph of a 4-km-long MR fibre wrapped on a spool. **g**, Side and cross-sectional views of the MR fibre. **h**, *A*_*y*_*/EI*_*y*_, serving as a criterion for identifying the yarn flexibility, of MR yarns with different fibre diameters and surface helical angles. Error bars correspond to s.d. (*n* = 3). **i**, Magnetization curves of the optimal MR yarn in the direction parallel and perpendicular to the yarn axis. **j**, Photograph of a knot formed by the MR yarn, demonstrating its flexibility and strength. Scale bars, 1 cm (**f**); 20 μm (**g**); 500 μm (**j**). Source Data

Yarn serves as an important intermediary linking the fibres to the final fabrics, fundamentally influencing the design, process and performance of the end textile products. To guide the strategic development of MR yarns, we developed a simple model that relates the bending and stiffening behaviour of MR yarns with the direction and strength of magnetic field vectors, considering both material properties and geometric structures of the yarn. It provided two indicators linked to those vector-stimuli-responsive behaviour: the ratio of material susceptibility (*χ*_m_) to Young’s modulus (*E*), *χ*_m_/*E*, for composite material development, and the ratio of cross-sectional area (*A*_*y*_) to the second moment of inertia (*I*_*y*_),* A*_*y*_*/I*_*y*_, for yarn geometric structure design (Supplementary Note 1 and Supplementary Figs. 1 and 2). This model facilitates a methodical approach, starting with the rational design of composite materials, processing through large-scale fabrication of MR fibres and culminating in the refinement of MR yarn geometric structures (Fig. 2a).

**Extended Data Fig. 1:**
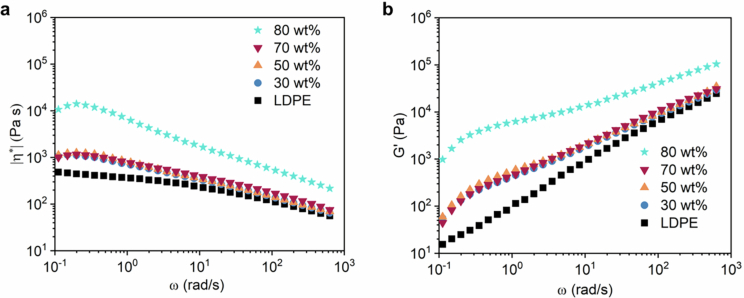
Rheology of molten LDPE and composites. **a**, Logarithmic plots of complex viscosity. **b**, Logarithmic plots of storage modulus as a function of angular frequency.

We rationally selected low-density polyethene (LDPE) as the polymer matrix and carbonyl iron particles (CIPs) as the magnetic filler to fabricate a series of polymer composites with uniform filler distribution by twin-screw extruder. We then systematically analysed their rheological characteristics to identify the melt-spinnable composite with the highest *χ*_m_/*E*. Rheological characteristics of the molten composites indicate good spinnability, demonstrating good extrusion flowability and high drawing stability for composites with CIPs up to 70 wt% (Fig. 2b, Extended Data Fig. 1 and Supplementary Note 2). The molten composite with 80 wt% CIPs exhibits solid-like behaviour (tan *δ* < 1) and plastic instability, posing difficulties to realize stable extrusion and drawing. In another aspect, increasing CIP content results in a more significant increase in *χ*_m_ than *E* (Supplementary Figs. 3 and 4), leading to higher *χ*_m_/*E* (Fig. 2c). Therefore, the composite containing 70 wt% CIPs with high *χ*_m_/*E* (1.23 emu cm^−3^ kOe^−1^ MPa^−1^ at 3 kOe) and good spinnability was finalized for MR fibre spinning.

**Extended Data Fig. 2:**
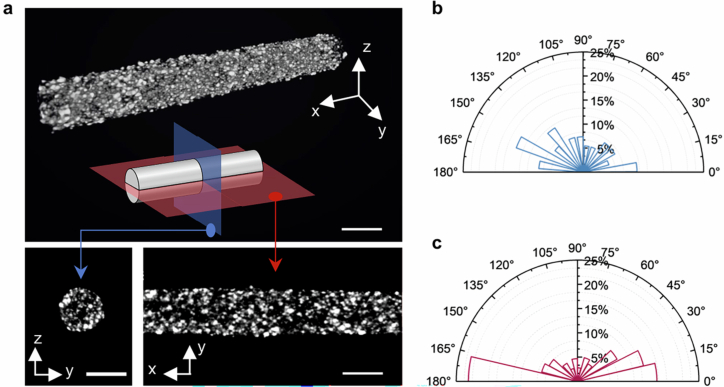
The distribution of CIPs in MR fiber. **a**, Nano-CT images of the MR fiber. Half-rose plots of the orientation of CIPs in **b**, Cross-sectional plane, and **c**, Longitudinal section plane. Scale bars, 50 μm.

**Extended Data Fig. 3:**
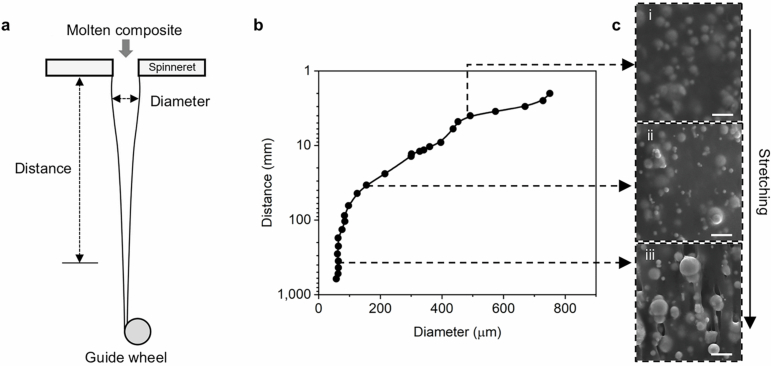
Evolution of MR fiber structure during melt spinning and in-situ high-speed drawing. **a**, Sketch of the single filament during the process of melt spinning and in-site high-speed drawing. **b**, Fiber diameter at different distances away from the spinneret. **c**, Surface morphology of fibers with diameters corresponding to those presented in **b**. Scale bars, 5 μm.

The optimal composite was processed into thin MR fibres with aligned polymer chains and CIP distribution by melt spinning and in situ high-speed drawing (Fig. 2a). After experiencing a high drawing ratio of 173, they were drawn down to a diameter of 57 μm (Fig. 2d and Supplementary Note 3). This extensive drawing not only stretched the polymer chains (Fig. 2d), increasing Young’s modulus and breaking strength of MR fibres (Fig. 2e and Supplementary Fig. 5), but also oriented the CIP distribution along the MR fibres (Extended Data Fig. 2), enhancing magnetic anisotropy of MR fibres. Furthermore, the drawing process moved CIPs out of the boundaryless surface (Extended Data Fig. 3), which in turn increased the surface roughness and the static coefficient of friction from 0.13 to 0.36 (Supplementary Fig. 6). The integration of melt spinning with high-speed drawing facilitated the continuous production of thin and high-loading MR fibres on a large scale, achieving a production speed of about 2 km h^−1^ (Fig. 2f,g).

**Extended Data Fig. 4:**
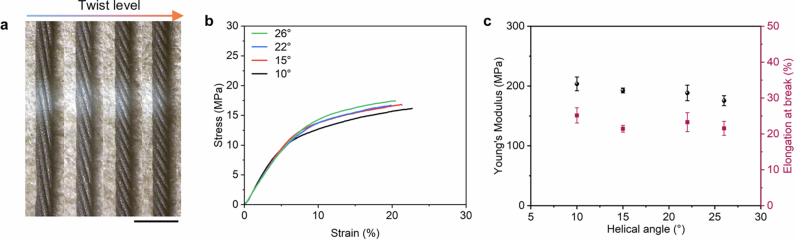
Mechanical properties of MR yarns with different twist levels. **a**, Photographs of MR yarns with different twist level, scale bar 500 μm. **b**, Stress-strain curves and **c**, The extracted Young’s modulus and elongation at break of MR yarns with different helical angles. Error bars correspond to s.d. (n = 3).

Seven MR fibres were twisted and subjected to heat-setting to produce MR yarns (Fig. 2a). Similar to *A*_*y*_*/I*_*y*_, *A*_*y*_*/EI*_*y*_ was found to inversely correlate the diameter of fibres and directly correlate the surface helical angle of yarn (Fig. 2h). Increasing the surface helical angle slightly influences the tensile characteristics of yarns (Extended Data Fig. 4). We limited it to 26° to reduce the risk of breakage during twisting. Consequently, we meticulously engineered the MR yarn to comprise seven fibres, each 57 μm in diameter, twisted to achieve a surface helical angle of 26°. This specific configuration resulted in a yarn with a high *A*_*y*_*/EI*_*y*_ (0.027 mN^−1^) and significant magnetic anisotropy (ratio of axial to radial magnetization 1.4 at 3 kOe; Fig. 2i). These characteristics are indicative of the bending and stiffening abilities of MR yarns. Moreover, the MR yarn demonstrated remarkable robustness and flexibility and can withstand 10,000 repeated bending and forming a tight knot without compromising its integrity (Fig. 2j and Supplementary Figs. 7 and 8), thereby making it suitable for standard textile processing.

## Characterization of MR yarns

**Fig. 3:**
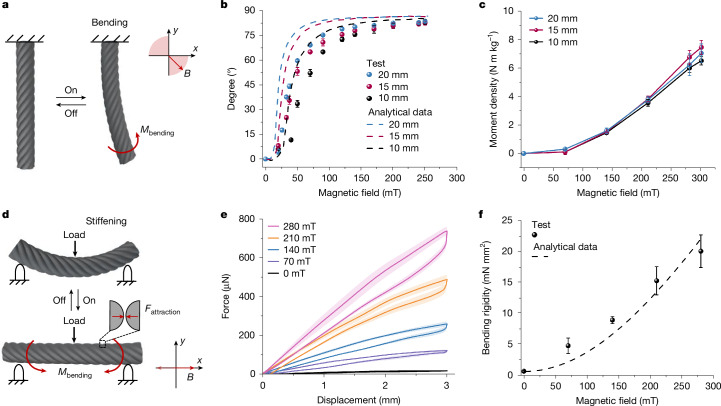
Bending and stiffening properties of MR yarns. **a**, Schematic of MR yarn undergoing bending actuation under a specific magnetic field. The yarn, cantilevered at one end, bends towards the direction of the magnetic field because of the angular misalignment between the length of the yarn and the magnetic field, generating a magnetic torque and a resultant bending moment (**M**_bending_). The yarn returns to its original vertical position on removal of the magnetic field. The range of the magnetic field direction is indicated in the related Cartesian coordinate system. **b**, Bending actuation angle plotted against the applied magnetic field strength and predicted from the bending actuation model for MR yarns with different lengths. Error bars correspond to s.d. (*n* = 3). **c**, Moment density of MR yarns with different lengths. Error bars correspond to s.d. (*n* = 3). **d**, Schematic of MR yarn stiffening under a specific magnetic field. With the yarn simply supported and under a central vertical load, applying a horizontal external magnetic field induces attraction (**F**_attraction_) between the wrapping MR fibres. This, along with any bending deformation, generates magnetic torques that straighten the yarn, resulting in reduced bending deformation compared with the conditions without a magnetic field. The range of the magnetic field direction is indicated in the related Cartesian coordinate system. **e**, Three-point bending force–displacement curves of MR yarns at different magnetic field strengths. Shaded areas correspond to s.d. (*n* = 3). **f**, Bending rigidity plotted against the applied magnetic field strength and predicted from the stiffening model for MR yarns. Error bars correspond to s.d. (*n *= 3). Source Data

The magnetized MR yarn consistently aligns its magnetic moment with the easy axis to endeavour the energetically favourable direction of magnetization. Figure 3a,d show that any angular misalignment between the easy axis and magnetic field induces magnetic torque. The resulting bending moment (**M**_bending_) either aligns the MR yarn with the field to reduce misalignment or opposes the external force to hinder further misalignment. Moreover, the attraction (**F**_attraction_) between wrapping MR fibres, induced through their demagnetization fields, contributes to the extra stiffening of MR yarn. These mechanisms induce two primary operation modes for MR yarns: bending mode and stiffening mode. As showcased in the comprehensive demonstration (Supplementary Video 1), when a vertical magnetic field is applied, a free-standing MR yarn promptly bends upwards, stiffens and stands upright. On removing the magnetic field, it swiftly returns to its original flexibility, becomes slack and lies down.

All the trends of MR yarns of various lengths aligned with the analytical predictions based on our MR yarn bending model (Fig. 3b), validating our assumption in modelling the yarn as a cylindrical rod (Supplementary Note 1 and Supplementary Fig. 9). Under the same magnetic field strength, the longer MR yarns generated larger bending degrees. The bending output of MR yarns was evaluated using the modified lifting test^42^. The moment densities of MR yarns with different lengths, characterized by moment per unit mass, were proportional to the magnetic field strength and reached about 7 N m kg^−1^ at 300 mT (Fig. 3c). Among these MR yarns with different lengths, the 10-mm-length MR yarn lifts 185 times of its body weight.

**Extended Data Fig. 5:**
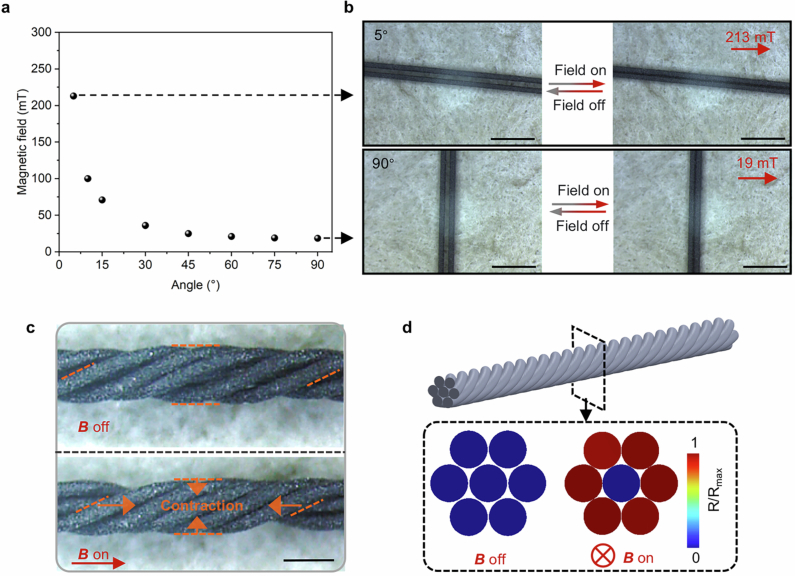
Magnetic interaction between MR fibers. **a**, Relationship between the minimal magnetic field strength required to bring parallel MR fibers together and the angle between the magnetic field direction and the fiber axis. **b**, Photos of the attraction between two parallel MR fibers after applying magnetic fields as related to **a**. Scale bars, 500 μm. **c**, The contraction of an MR yarn with 1 core and 5 wrapping MR fibers under a lengthwise magnetic field. Scale bar, 200 μm. **d**, Finite element analysis results identifying the compacting of an MR yarn under a lengthwise magnetic field, where *R* and *R*_max_ represented the displacement and maximum displacement of wrapping fibers in the radial direction, respectively.

The stiffening property of MR yarns under parallel magnetic fields was assessed by three-point bending with a 15-mm gap. The bending force increased with displacement and magnetic field strength (Fig. 3e). As the magnetic field rose from 0 mT to 280 mT, the bending rigidity of MR yarn increased by about 30 times, from 0.68 mN mm^2^ to 20 mN mm^2^, aligning well with our analytical prediction (Fig. 3f). This remarkable tunability in stiffness is primarily attributed to two mechanisms. First, the magnetic torque, a known effect in similar anisotropic soft magnetic structures, plays a significant part. Furthermore, we identified an unusual mechanism: an increase in internal friction caused by magnetic attraction among helical fibres within the magnetized MR yarn, which enhances its bending stiffness (Extended Data Fig. 5, Supplementary Note 4, Supplementary Fig. 10 and Supplementary Video 2).

We compared the performance, including safety, bending angle, moment density, stiffening window and maximum flexibility of the MR yarn, with other yarn and fibre materials that demonstrate bending or stiffening properties reported recently (Supplementary Table 1). The MR yarn uniquely combines both bending and stiffening in a single material, unlike others that require multiple activatable components within a single fibre to achieve both functions. Its high flexibility far surpasses that of conventional yarns and fibres, which often use thicker fibres with diameters of several hundred to thousands of micrometres. Together with high magnetic anisotropy, the MR yarn demonstrates superior bending actuation properties. Moreover, it offers a nearly 30-fold tunable stiffness range within safe magnetic field intensities, making it ideal for wearable smart textiles. Although similar stiffening ranges can be achieved with thermal-responsive fibres, their high operating temperatures raise safety concerns about skin contact (Supplementary Note 5).

## Characterization of MR fabrics

**Fig. 4:**
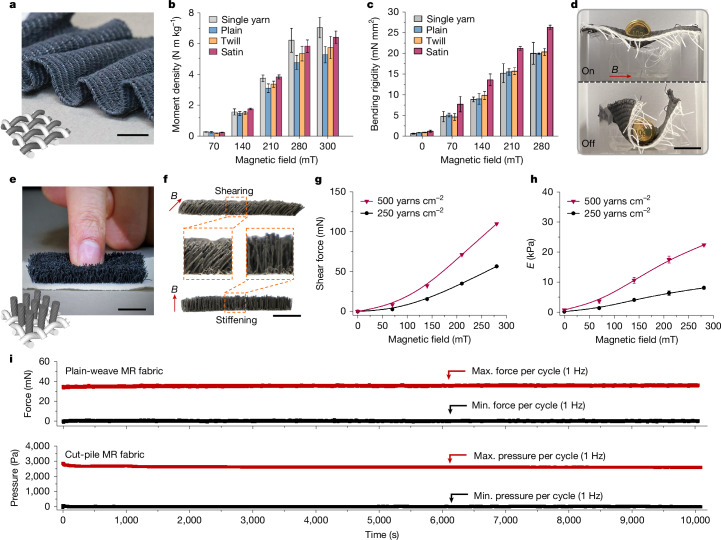
Structure and property of woven and cut-pile MR fabrics. **a**, Photograph of a flexible plain-weave MR fabric with structure diagram at bottom left. **b**, Moment density of single MR yarn and plain-, twill- and satin-weave MR fabrics. Error bars correspond to s.d. (*n* = 3). **c**, Bending rigidity of single MR yarn and plain-, twill- and satin-weave MR fabrics normalized per MR yarn. Error bars correspond to s.d. (*n* = 3). **d**, Photographs of a plain-weave MR fabric with the stiff–soft duality. **e**, Photograph of a soft and conformable cut-pile MR fabric with structure diagram at bottom left. **f**, Side-view photographs of the cut-pile MR fabric showing in-plane shearing and out-of-plane stiffening under specific magnetic field directions. **g**,**h**, Shear force (**g**) and compression modulus (**h**) of cut-pile MR fabrics with specific yarn densities. Error bars correspond to s.d. (*n* = 3). **i**, Compression fatigue test on MR fabrics at a frequency of 1 Hz with an extension of 1.5 mm for more than 10,000 consecutive cycles, under specific magnetic fields with a strength of 140 mT. Scale bars, 10 mm (**a**,**d**,**e**,**f**). Source Data

**Extended Data Fig. 6:**
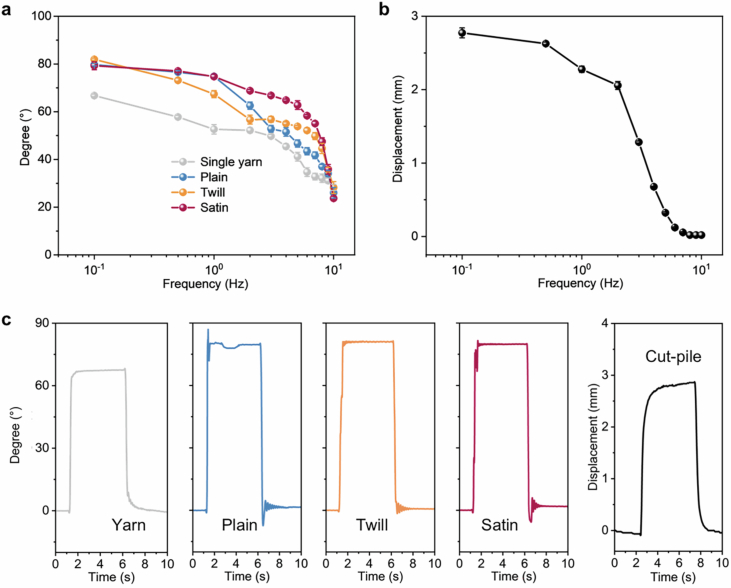
Dynamic actuation properties of MR fabrics and yarn. **a**, Bending actuation angles of the MR yarn and woven fabrics under square wave actuation at specific frequencies with a peak magnetic field strength of 135 mT. Error bars correspond to s.d. (n = 3). **b**, In-plane displacement of the MR yarn tip of cut-pile MR fabric under square wave actuation at specific frequencies with a peak magnetic field strength of 247 mT. Error bars correspond to s.d. (n = 3). **c**, Bending angle with time of MR yarn and woven fabrics under square wave actuation with a peak magnetic field strength of 135 mT. In-plane displacement with time of MR yarn tip of cut-pile MR fabric under square wave actuation with a peak magnetic field strength of 247 mT.

To scale up the moment output and bending rigidity as well as enable practical functionalities, MR yarns were efficiently assembled into woven and cut-pile MR fabrics in parallel and vertical orientations, respectively. Thin and flexible woven MR fabrics were created by interlacing MR yarns and sewing thread at the right angle (Fig. 4a). The moment density reaches a maximum when a straight yarn was aligned at 45° to the magnetic field (Supplementary Fig. 11 and Supplementary Note 1, Eq. (7)). However, after interlacing the yarns into woven fabrics, the moment density slightly decreases (Fig. 4b). This reduction results from the crimped structure of interlaced yarns that causes small deviations in the orientation of yarn segments from the optimal 45° alignment with the magnetic field. For the plain, twill and satin weaves we fabricated, the weave angle varies inversely with float length, resulting in a statistically significant correlation between float length and moment density at magnetic field strengths between 140 mT and 300 mT (Supplementary Note 6 and Supplementary Fig. 12). The MR yarn and woven fabrics retain at least 30% of their bending degree as the actuation frequency increases from 0.1 Hz to 10 Hz, and exhibit fast response times ranging from 0.07 s to 0.22 s and settling times between 0.52 s and 0.62 s (Extended Data Fig. 6a,c and Supplementary Note 7).

The bending rigidity normalized per MR yarn of woven MR fabrics is slightly higher than that of the single MR yarn and increases with float lengths (Fig. 4c). This increase is due to the higher MR yarn packing density, lifting constraint and friction experienced by the MR yarns during bending deformation, which ultimately increases the energy dissipation (Supplementary Fig. 13). Among the MR fabrics, the plain-weave MR fabric mostly resembles the MR yarn because of the lowest level of interaction between adjacent MR yarns. A flexible plain-weave MR fabric measuring 35 × 40 mm and weighing 0.5 g supports a 10 g weight at a magnetic field strength of 210 mT (Fig. 4d and Supplementary Video 3).

In comparison with woven MR fabrics consisting of in-plane MR yarns, a cut-pile MR fabric consists of MR yarns vertically inserted into the base fabric. It is soft and conformable (Fig. 4e) and can output shear displacement and force and tune compression rigidity in response to specific external magnetic fields (Fig. 4f). The cut-pile MR fabric generates in-plane shear force when subjected to a magnetic field inclined to the plane. All the bending actuation of MR yarns in the same direction is converted into the directional movement of the surface plane made up of MR yarn tips. The actuation displacement responds quickly within 0.23 s and settles at 1.87 s on the application of magnetic fields, maintaining a displacement of above 2 mm under dynamic actuation at frequencies of up to 2 Hz (Extended Data Fig. 6b,c and Supplementary Note 7). The in-plane displacement results in a global shear force that is proportional to the magnetic field strength and the MR yarn density (Fig. 4g). A 1 cm^2^ area of cut-pile MR fabric with a density of 500 yarns cm^−2^ can generate 110 mN of shear force when exposed to a static magnetic field of 280 mT with an angle of 45° to the plane.

Moreover, the cut-pile MR fabric can tune the out-of-plane compression modulus by varying the strength of the magnetic field applied perpendicular to the plane. Each magnetized MR yarn resists axial compression because the buckling deformation misaligns the MR yarn with the magnetic field. The sum of the force of MR yarns in the axial direction is shown as the apparent compression force of the cut-pile MR fabric in its thickness direction (Supplementary Fig. 14). The absolute range of the compression modulus is customizable by changing the MR yarn density. Doubling the MR yarn density from 250 yarns cm^−2^ to 500 yarns cm^−2^ adjusts the modulus range from 0.45–8.3 kPa to 1–22.5 kPa (Fig. 4h). Both the woven and cut-pile MR fabrics are durable to preserve stiffening actuation after a continuous fatigue test of about 10,000 cyclic compression (Fig. 4i) and exhibit minimal creep deformation under consistent loads for 1,000 s, with and without magnetic stiffening (Supplementary Fig. 15).

## Smart textile demonstrations

**Fig. 5:**
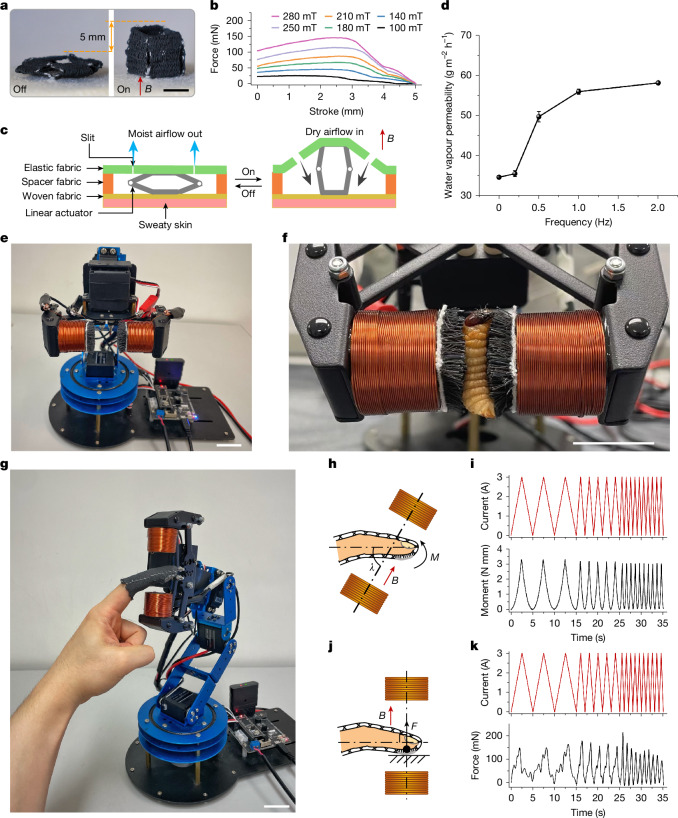
Demonstration of smart textiles based on MR fabrics. **a**, Fabric linear actuator with 5 mm stroke enabled by woven MR fabrics in a four-sided Sarrus linkage. **b**, Force–stroke relationship of the actuator under specific magnetic field strengths. **c**, Structure and working principle of the active ventilation fabric driven by the linear actuator. **d**, Water vapour permeability of the active ventilation fabric under square-wave actuation at selected frequencies with a peak field of 250 mT. Error bars correspond to s.d. (*n* = 3). **e**, Conformal gripper using cut-pile MR fabrics on coaxial electromagnets mounted on a robotic arm with six degrees of freedom. **f**, Close-up of the gripper holding a live worm (1 A input current). **g**, Untethered haptic finger glove remotely controlled by the mobile magnetic actuation system for haptic feedback. **h**, Schematic showing the positional and orientational relationship between the finger wearing the MR finger glove and the coaxial electromagnet pair for kinaesthetic feedback. Misalignment between the magnetic field and the finger axis, defined by angle *λ*, generates a moment (**M**) on the MR woven fabric. **i**, At *λ* = 60°, triangular currents (3 A peak) at 0.2 Hz, 0.5 Hz and 1 Hz were applied to the electromagnet pair, and the resulting output moment of the MR finger glove was measured. **j**, Schematic showing the MR finger glove with the fingertip pressing on a flat surface to mimic touch. A magnetic field applied perpendicular to the finger axis activates the cut-pile MR fabric under the fingertip, producing a normal force (**F**). **k**, At *λ* = 90°, triangular currents (3 A peak) at 0.2 Hz, 0.5 Hz and 1 Hz were applied to the electromagnet pair, and the resulting output force of the MR finger glove was measured. Scale bars, 5 mm (**a**); 2 cm (**e**,**f**,**g**). Source Data

The functionalities and performance of MR fabrics enable a wide range of applications. We designed a linear fabric actuator that operates by converting bending moments into linear motion (Fig. 5a). We fabricated an instantiation of fabric linear actuator that generates a stroke of 5 mm and a force of 150 mN at a magnetic field strength of 280 mT (Fig. 5b). Furthermore, we sandwiched this linear actuator between two fabrics to create an active ventilation fabric (Fig. 5c). Cyclic activation of the linear actuator by a stationary electromagnet induces a fluttering motion of the elastic fabric along with periodic opening of slits, promoting air exchange of the micro-environment (Supplementary Video 4). The breathability of the active ventilation fabrics, characterized by the water vapour transmission rate, is efficiently controlled between 34.5 g m^ −2^  h^−1^ and 58.5 g m^−2^ h^−1^ by operating at frequencies varying from 0 Hz to 2 Hz (Fig. 5d and Supplementary Fig. 16), offering potential benefits in personal moisture and thermal management.

We designed an integrated conformable gripping device by symmetrically integrating cut-pile MR fabrics onto the poles of a mobile magnetic actuation system (Fig. 5e, Supplementary Figs. 17 and 18 and Supplementary Note 9). An array of independently axial compressible MR yarns with tunable modulus enables the cut-pile MR fabrics to have more effective adaptability to irregular shapes with maximized contact area to evenly distribute the gripping force applied on the objects than a conventional cushion. Thus, the conformable gripping device with high workspace flexibility effectively grasps and transfers different items, such as worms, tofu, blueberries, mung bean cake, potato chips and fusilli, with a wide range of modulus at minimal risk of damage or deformation (Fig. 5f and Supplementary Video 5).

Finally, we demonstrated a remote-controllable haptic device by leveraging the mobile magnetic actuation system to activate the finger glove that consists of cut-pile and plain-weave MR fabrics, ensuring a lightweight, comfortable wearing experience (Fig. 5g). This haptic finger glove provides a variety of haptic sensations, including kinaesthetic and tactile effects by controlling the mobile magnetic actuation system to modulate the spatial relationship between the electromagnets and the finger glove and adjust the electric current parameters (Fig. 5h,j, Supplementary Figs. 19–22, Supplementary Note 10 and Supplementary Videos 6 and 7). For instance, for a current of 3 A, the finger glove generates a moment of approximately 3.5 N mm, translating to a moment density of 2.6 N mm g^−1^ on the finger by the plain-weave MR fabric, whereas it produces a normal force of 150 mN to the fingertip pad (0.7 cm^2^) by the cut-pile MR fabric (Fig. 5i,k). These performances are comparable to those of the commercial Dexmo glove (9.5 N mm g^−1^) (refs. ^43,44^) and meet the typical threshold of human tactile perception in the order of 10 mN (ref. ^45^). It can be controlled to change the softness and smoothness sensations felt by the fingertip, showing great protentional as a fabric handle emulator for rendering the hand feel of different fabrics. Unlike conventional kinaesthetic gloves that rely on motors or pumps, our finger glove based on MR yarns eliminates the complex transmission mechanisms and the need for grounding, resulting in a more lightweight, less-restrictive and more natural design for the wearing finger.

## Conclusions

In summary, we have developed an engineering guide for multi-hierarchy fibrous actuating structures by integrating the structural mechanics of textiles with the magnetics of soft magnetic materials. Using a scalable fabrication process, we have created vector-stimuli-responsive MR fibrous materials with various levels of hierarchy architectures. The resultant km-long continuous MR fibres, with a diameter of 57 μm and a particle load of 70 wt%, exhibit excellent alignability subject to an external weak and human-safe magnetic field of up to 300 mT. The MR yarns made from these fibres demonstrate an outstanding bending moment density of 6.5 N m kg^−1^ and an extraordinarily wide stiffness regulation range of 30 times as compared with other stimuli-responsive materials. Furthermore, we have constructed woven and cut-pile MR fabrics from the yarns. The resultant MR fabrics illustrate a diverse array of actuation abilities, including bending, shearing and linear motion, as well as stiffening properties under bending and compression. Finally, we showcase the versatility of these smart textiles through several demonstrations: an active ventilation fabric, an integrated adaptable gripping device and an untethered all-fabric haptic finger glove. This work not only advances the field of stimuli-responsive materials but also opens up possibilities for the practical application of smart textiles in everyday life and various industries.

## Methods

### Preparation of MR yarns

**Extended Data Fig. 7:**
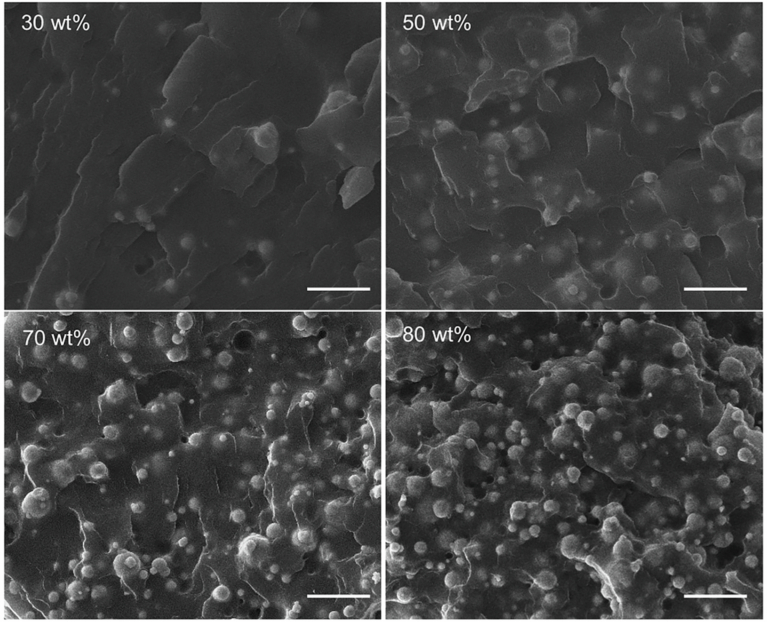
The dispersion of CIPs in bulk composites. SEM images of fracture surfaces of the composites with CIP contents of 30, 50, 70, and 80 wt%. Scale bars, 10 μm.

We selected LDPE as the flexible matrix because of its high filler capacity enabled by highly branched chains with low molecular packing^46^ and good flowability indicated by a broad molecular weight distribution (Supplementary Fig. 23) and significant shear thinning (Extended Data Fig. 1a). CIP was chosen over other soft magnetic materials for its high susceptibility and low remnant magnetization, availability in micro-spherical particles, cost-effectiveness and resistance to oxidation with SiO_2_ coating (Supplementary Table 2). CIPs coated with SiO_2_ (SQ, BASF SE; Supplementary Fig. 24) were dispersed within LDPE (1700 MN 18C, total energies SE; melt flow rate of 70 g/10 min at 190 °C/2.16 kg) by melt compounding by a twin-screw extruder (Thermo Fisher Hot Melt Extruder Pharma 11). The temperature profile, ranging from the hopper to the die, was set at 10, 80, 130, 150, 150, 150, 150, 150, and 150 °C, respectively, while the screw speed was kept at 50 rpm. Four LDPE/CIP composites were prepared, varying in CIP content at 30, 50, 70, and 80 wt%. Interfacial interactions between LDPE molecules and CIPs (Supplementary Note 8 and Supplementary Fig. 25) promote the wetting of polymer on CIP surfaces, ensuring the uniform CIP dispersion of composites with filler content up to 80 wt% (Extended Data Fig. 7). Among these, the composite containing 70 wt% CIPs was chosen for fibre spinning.

This selected composite was introduced into the barrel of a laboratory melt spinning machine (AT225, Anytester Hefei) to produce MR fibres. The three heating zones of the barrel were set at 140 °C, 150 °C and 160 °C, respectively, allowing the composites to melt for 10 min until reaching a stable temperature of 160 °C. Nitrogen was then filled into the barrel to provide a pressure of 1.2 MPa. The molten polymer strands were extruded through a spinneret and guided by a ceramic wheel onto a collection roller with a diameter of 8.2 cm. A cooling fan positioned between the spinneret and the guide wheel facilitated solidification of the molten composite fibres. The diameter of the resulting MR fibre could be controlled by adjusting the winding speed, and in this case, the extruded molten filaments with a diameter of 750 μm underwent rapid thinning over a short range of about 20 cm (Extended Data Fig. 3). MR fibres with a diameter of 57 μm were consistently produced at a winding speed of 130 rpm. Then, seven of these fully drawn fibres were twisted clockwise to form a yarn, which was subsequently heat-set in an oven at 60 °C for 1 h to alleviate residual stress and stabilize the twist configuration. The primary goal in optimizing the structure of MR yarn was to maximize *A*_*y*_/*I*_*y*_, which is inversely related to the diameter of the fibres and directly related to the helical angle of the yarn (Supplementary Note 1). Given that the fibre diameter directly correlates with its modulus, our evaluation focused on optimizing *A*_*y*_/*EI*_*y*_ to refine the MR yarn design.

### Fabrication of MR fabrics

**Extended Data Fig. 8:**
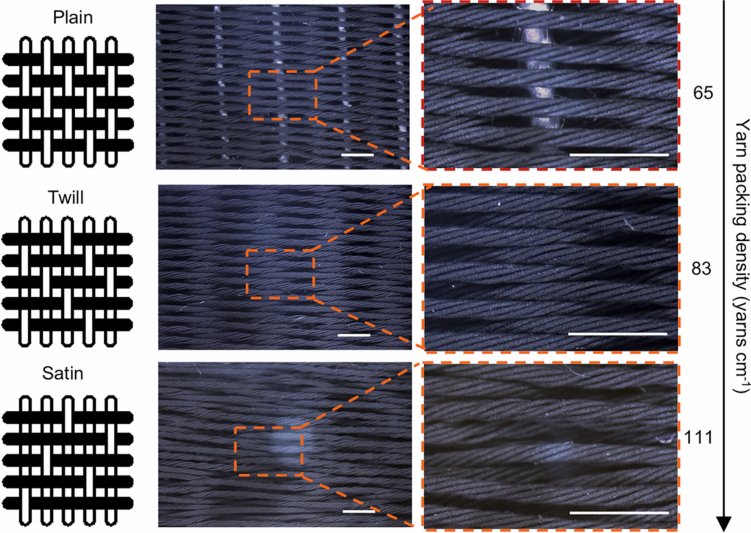
Structure of woven MR fabrics. Weave diagrams of plain, 2/1 twill, and 4/1 satin MR fabrics. Black and white fill represents weft and wrap, respectively. Photographs with zoom-in views of corresponding fabrics are arranged at the right side. The MR yarns are interlaced as weft with different packing densities along the wrap direction. Scale bars, 1 mm.

Woven MR fabrics were fabricated by interlacing MR yarns as weft and sewing thread as warp at right angles, using a hand weaving machine. Float lengths of 1, 2 and 4 were specifically chosen to fabricate plain-, twill-, and satin-weave MR fabrics that exhibit packing densities of 65 yarns cm^−1^, 83 yarns cm^−1^, and 111 yarns cm^−1^, respectively (Extended Data Fig. 8). These selections enable the examination of the spatial freedom of interlacing MR yarns concerning their float length and packing density. The plain weave has the highest number of interlacing points, followed by the twill weave, and the satin weave.

Cut-pile MR fabrics were fabricated by inserting MR yarns into holes of a base plain-weave fabric using a punch needle kit. On pulling the needle out, a loop of MR yarn formed on the opposite side, its length controlled by the depth of needle insertion. This process repeated until the predefined area was uniformly filled at yarn densities of 250 yarns cm^−2^ or 500 yarns cm^−2^. Subsequently, a thin layer of silicone glue was applied to secure the inserted yarns. The loops were then cut at their centre points, followed by the application of silicone precursor at the cut tips. After curing overnight at room temperature, the silicone effectively prevented the free ends from untwisting.

### Coefficient of friction

The as-spun filaments, each with a length of 3 cm, were arranged in parallel without any gaps to form a 1-cm wide region and adhered onto a square glass measuring 3 cm × 3 cm and weighing 1.8 g using a double-sided tape. Two identical samples were prepared for the measurement: one with the filaments facing upwards, and the glass side fixed onto a horizontal linear platform, and the other sample with the filaments facing downwards, aligned face-to-face and parallel to the filament region of the first sample. At the left edge of the top glass packed with filament tips, the centre point of the glass edge was horizontally connected to a force gauge using a nylon filament. A series of weights (1 g, 2 g, 5 g, 10 g, 20 g, 50 g and 100 g) were separately placed on top of the sample to provide normal force *F*_nf_ *= m*_tm_*g*, where *m*_tm_ is the total mass of the weight and top glass substrate, and *g* is the acceleration due to gravity. The linear platform moved away from the force gauge at a velocity of 5 µm s^−1^. The maximum reading of the force gauge was recorded as the maximum static friction *F*_friction_, and the static friction coefficient *a* was calculated using the formula: *F*_friction_* = aF*_nf_. The static coefficient of friction between MR fibres was measured in the same way, by replacing the filaments with fibres.

### Differential scanning calorimetry

Differential scanning calorimetry experiments were conducted using a Mettler Toledo DSC3 in a nitrogen atmosphere. Each sample, weighing approximately 1.5 mg, was analysed within a temperature range of 55–135 °C at a heating rate of 10 °C min^−1^ to record the endothermic curves. The heat of fusion (Δ*H*_f_) was determined by integrating the heat flow between 60 °C and 115 °C. This parameter was then used to calculate the crystallinity degree (*χ*) of both pure LDPE and the composite matrix. The crystallinity degree *χ* was defined as the ratio of Δ*H*_f_*/*(1 − *x*), where *x* represents the content of CIPs in the composite, to the heat of fusion (289.9 J g^−1^) of the purely crystalline form of polyethylene^47^.

### MR fibre thermal shrinkage

MR fibre shrinkage was assessed by subjecting the MR fibres with a length of 30–50 mm to a temperature of 100 °C for 10 min without external constraints. Shrinkage was quantified as the percentage change in the original MR fibre length using the formula, Shrinkage (%) = 100 × (*L*_if_ − *L*_sf_)*/L*_if_, where *L*_if_ represents the initial fibre length and *L*_sf_ represents the fibre length after complete shrinkage. The thermal shrinkage results provide insights into both the elongation and arrangement of polymer chains within the MR fibres. This is attributed to the relaxation of polymer chains during the thermal shrinkage process.

### Tensile measurement

LDPE and composites containing 30 wt%, 50 wt% and 70 wt% CIPs samples with a dimension of 30 × 10 × 0.105 mm^3^ were stretched to break at a velocity of 0.3 mm s^−1^ by a universal testing machine (Model 5566, Instron).

### Structure characterization

Scanning electron microscopy images were obtained using a Tescan VEGA3 microscope equipped with an energy dispersive X-ray spectroscopy detector. Polymer composite samples were subjected to freeze fracture in liquid nitrogen to expose the cross-sectional surfaces, and then sputter coating was done with Au. The imaging was conducted at an accelerating voltage of 20 kV. The energy dispersive X-ray spectroscopy mapping was performed to analyse the distribution of Si, O and Fe on the surface of CIP. Optical photographs were captured using a Leica M165 C High-Performance Stereo Microscope to visualize the structure and extract the geometrical parameters of yarns and fabrics. Nano-computed tomography analysis was conducted using a Zeiss Xradia 520 Versa 3D X-ray microscope to examine the distribution of CIPs within the MR fibre. An MR fibre, approximately 1 mm in length, was mounted on the sample stage. Cross-sectional images, each with a resolution of 480 nm, were sequentially captured along the length at specified intervals. These images were then processed to generate a 3D reconstruction of the MR fibre. The polymer matrix was then extracted, in which the white zones represent CIPs. The directional distribution of CIPs within the MR fibre was assessed using ImageJ software with the following processing steps^48^. Initially, the raw nano-computed tomography images of both transverse and longitudinal sections were converted into binary images using an appropriate threshold to delineate the CIP distribution. Subsequently, the adjustable watershed algorithm was applied to segment the binary image effectively. The areas reflecting concentrated CIPs appeared as white zones within the images and were then fitted with ellipses to account for any orientation in the CIP distribution. Finally, the orientation of each CIP-concentrated area was determined by measuring the tilt angles of the ellipses relative to the horizontal plane. The degree of orientation was analysed based on the percentage distribution of different angles.

### Magnetization characterization

Magnetic measurements were conducted at room temperature using the Physical Properties Measurement System (Quantum Design) with a sweep of external magnetic field ranging from −13 kOe to 13 kOe. Samples, including CIPs, LDPE, and composites with CIP contents of 30 wt%, 50 wt%, and 70 wt%, were tested without any preferred direction. The MR yarn was tested twice with the axis oriented parallel and perpendicular to the direction of the applied magnetic field.

### Oscillatory shear rheology

Dynamic rheological characterization of both neat LDPE and composites was conducted using a rheometer (AR 2000EX, TA Instruments) equipped with parallel plate geometry. The samples were compressed into disc-shaped specimens with a thickness of 2 mm and a diameter of 25 mm for testing purposes. Frequency sweeps were performed at 160 °C, with the frequency ranging from 100 Hz to 0.01 Hz, while maintaining a strain of 0.5% within the linear viscoelastic region.

### Dynamic mechanical analysis

Dynamic mechanical analysis experiments were carried out using a Mettler Toledo DMA1. Samples of LDPE and composites containing 70 wt% CIPs were analysed from −150 °C to −50 °C at a heating rate of 5 °C min^−1^ to record tan *δ* curves.

### Bending actuation characterization

The bending property of MR yarns was characterized by subjecting vertically cantilevered MR yarns, anchored at their top end, to uniform magnetic fields. MR yarns were cantilevered at the top and positioned vertically within the central space between the two poles of an electromagnet (PEM-20, Litian Magnetoelectrican Science & Technology). The desktop electromagnet measures 260 mm (length) × 180 mm (width) × 200 mm (height) with two 40 × 40 mm poles spaced at an adjustable distance of 0–60 mm (Supplementary Fig. 26a). Within the space between poles, highly uniform magnetic fields can be generated with controllable strength by varying the current and the pole distance (Supplementary Fig. 26b–e). This stationary device allows for precise and uniform magnetic field adjustments across a wide range, providing a versatile workspace for material property characterization and testing in various applications. Static magnetic fields were oriented at 87° to the yarn axis to control the direction of bending; as the magnetic field strength increased, the cantilevered MR yarns gradually bent upwards (Supplementary Fig. 27a). The bending deformation was captured using a camera, and the images were processed using ImageJ to quantify the bending degree. MR yarns with lengths of 10 mm, 15 mm and 20 mm were tested.

To measure the moment output of a MR yarn with length *L*_yarn_ and weight *m*_yarn_, a thin paperboard strip, of weight *m*_ps_, was attached along the entire free portion of the cantilevered MR yarn (Supplementary Fig. 27b). The magnetic field strength was set to specific values (70 mT, 140 mT, 210 mT, 280 mT and 300 mT), and the cantilevered MR yarn lifted until the midpoint was blocked perpendicular by the PMMA rod connected to the force gauge. At this point, the yarn formed a 45° angle with the vertical direction. The force *F*_block_ required to block the midpoint was recorded. The moment density of the MR yarn was calculated as *M*_pm _*= M*_bending_/*m*_yarn_ = [(*m*_ps_/*m*_yarn_ + 1)*g* sin 45° +* F*_block_]*L*_yarn_/2. The output moment per unit mass of fabrics was measured in a similar way by using woven MR fabrics with float lengths of 1, 2 and 4 measuring 20 × 5 mm with MR yarns aligned along the length direction. For each float length, three independently fabricated samples were evaluated separately.

### Stiffening characterization

The MR yarns with a length of 25 mm were measured by a three-point bending test (Supplementary Fig. 28). The lower anvil with a gap of 15 mm was fixed onto an acrylic tube, which was placed on the pan of an analytical balance (ME204, Mettler Toledo) to record the force. To prevent magnetic interference with the balance, a PMMA support with a height of 25 cm was placed beneath the three-point bending support anvil. The upper anvil was connected to an Instron, which provided a downward displacement of 3 mm and returned to the starting position at the rate of 0.1 mm s^−1^. Three samples of each type of yarns were tested separately. Magnetic fields were generated by a pair of N52 cylindrical magnets (diameter 70 mm and thickness 30 mm) placed parallel to the opposite poles face to face. The magnetic field strength was controlled by the distance between the two magnets. The woven MR fabrics were tested in a similar way by using a load cell (2530-10N) for recording the force.

### Bending durability test

The bending durability of the MR yarn was evaluated using a continuous bending setup (Supplementary Fig. 29). A 30-cm sample was fixed at one end to a vertical glass surface. The yarn was threaded horizontally through two pairs of guide pulleys (diameter 13.76 mm) spaced 8 cm apart, followed by a single pulley (diameter 17.2 mm) mounted on a PMMA plate. A pre-tension of 32.7% of the breaking strength of the yarn was applied to the free end using a hook-weight assembly. A pair of guide pulleys (diameter 13.76 mm) was attached to a moving PMMA fixture on an Instron testing machine, programmed to cyclically bend the yarn 90° upwards and 90° downwards for around 5,000 cycles at 0.25 Hz.

### Creep test

A plain-weave MR fabric (35 mm × 40 mm) with MR yarns aligned lengthwise was fixed at both ends with a 30-mm gap. A 100-g load on a PMMA block (3.25 g) applied a uniform pressure of 2.95 MPa over an area of 35 mm × 10 mm across the fabric centre. Initial deflection on loading was 0.84 mm, with no observable creep within 1,000 s. Under a magnetic field of 178 mT along the MR yarn axis, the initial deflection of the fabric decreased because of magnetic stiffening, with minimal creep observed over the same period.

A cut-pile MR fabric (40 mm × 40 mm) with a 4-mm-thick MR yarn layer was uniformly pressed with a pressure of 0.71 MPa by a loading of 113.6 g on the MR yarn layer, its thickness decreased to 3.65 mm. No additional thickness reduction occurred across 1,000 s, and under a vertical magnetic field of 187 mT, the initial compression decreased with no detectable creep on this timescale.

### Finite-element analysis

A 3D model was constructed using the Magnetomechanical node of COMSOL Multiphysics. Two rectangular magnets measuring 300 μm × 300 μm × 50 μm were built face to face to provide uniform magnetic fields. The surrounding air domain is modelled as a sphere with a radius of 2,300 μm. The two permanent magnets and air domain were from the COMSOL material library, and the mechanical and magnetic properties of the MR yarn were entered based on the experimental results.

### Linear actuator fabrication and test

The basic concept uses radially symmetric woven MR fabrics, each resembling a hinge, with a shared wrap yarn acting as a pivot to couple axial force to the bending moments of the woven MR fabrics. The linear fabric actuator was fabricated by integrating four plain-weave MR fabric hinges in the form of a four-sided Sarrus linkage. Each plain-weave MR fabric hinge was assembled by two plain-weave MR fabrics, measuring 5 mm × 5 mm, which shared the same wrap yarn as a pivot. To measure its stroke and force output, the linear actuator was placed on a PMMA plate within a vertical magnetic field (Supplementary Fig. 30). Initially, the actuator was fully extended, with the horizontal part of the PMMA fixture touching its top surface without force. An Instron machine then applied a downward displacement of 5 mm at 0.25 mm s^−1^, pressing the actuator down and recording its upward force on the PMMA fixture. After each test, the PMMA fixture returned to the starting position by the Instron machine. At the same time, the linear actuator also returned to the starting configuration due to the magnetic actuation.

### Ventilation fabric demonstration

The linear actuator was sandwiched in the centre between an elastic fabric with slits and a breathable woven fabric, with an annulus of spacer fabric stitched at the edge to form a dis-shaped active ventilation fabric with a diameter of 90 mm and a thickness of 5 mm.

The water vapour permeability (WVP) of active ventilation fabric was measured according to BS 7209. The active ventilation fabric with elastic fabric facing upwards was firmly fixed on top of a glass vessel (diameter 70 mm) filled with about 30 g distilled water and operated under square-wave actuation at frequencies of 0 Hz, 0.2 Hz, 0.5 Hz, 1 Hz and 2 Hz with a peak magnetic field strength of 250 mT for 30 min. The WVP is calculated as WVP = *M*/(*At*), where *M* is the water loss in mass, *t* is the time duration and *A* is the area of the exposed fabric.

## Online content

Any methods, additional references, Nature Portfolio reporting summaries, source data, extended data, supplementary information, acknowledgements, peer review information; details of author contributions and competing interests; and statements of data and code availability are available at 10.1038/s41586-025-09706-4.

## Supplementary information

Supplementary Information This file contains Supplementary Notes 1–10, Supplementary Figs. 1–30, Supplementary Tables 1 and 2, and references.

Supplementary Video 1 MR yarn stands up and lays down.

Supplementary Video 2 Attraction between magnetized wrapping MR fibres. An MR yarn comprising five slightly separated helical MR fibres wrapped around a core MR fibre was laying horizontally. When a magnetic field was applied along the yarn axis, the five helical MR fibres attracted each other. Once the magnetic field was removed, the fibres separated and returned to their original relative positions.

Supplementary Video 3 Fast and sufficient stiffness change of MR woven fabric. The flexible fabric was positioned horizontally on a PMMA supporter, and upon applying a magnetic field, it aligned with the field direction and remained flat. A 10 g load applied at the fabric centre caused a minor deflection, leading to misalignment of the MR yarns with the magnetic field. This deformation created magnetic moments in the fabric to balance the weight. Once the magnetic field was removed, the fabric lost the magnetic moments and could not support the weight, causing it to return to its flexible state and release the load.

Supplementary Video 4 Active ventilation fabric in operation. The left section shows the full view of the ventilation fabric device in action. A stationary electromagnet applies a dynamic magnetic field at different frequencies to the fabric. This causes the linear actuator within the fabric to push the elastic fabric upward when the magnetic field is active. When the field is off, the elastic fabric returns to its flat state due to its own deformation force. The right section offers a close-up view of how the elastic fabric with slits deforms during this dynamic actuation.

Supplementary Video 5 Demonstration of the conformable gripper. Integrated conformable gripping device for handling delicate items with varying shapes, surface conditions and stiffness.

Supplementary Video 6 Kinesthetic feedback generated by remote-controllable haptic finger glove. The haptic MR finger glove was worn on a silicone finger. Dynamic magnetic fields were applied to the glove through a mobile magnetic actuation system, producing various kinesthetic effects. This untethered device demonstrated high feasibility by allowing precise control over the rotation speed and angle of the magnetic field direction relative to the finger axis, as well as adjustments to the current waveform, amplitude and frequency.

Supplementary Video 7 Tactile feedback generated by remote-controllable haptic finger glove. The haptic MR finger glove was worn on a silicone finger. Dynamic magnetic fields were applied to the glove through a mobile magnetic actuation system, producing various haptic effects. This untethered device demonstrated high feasibility by allowing precise control over the current waveform, amplitude and frequency.

## Source data

Source Data Fig. 2

Source Data Fig. 3

Source Data Fig. 4

Source Data Fig. 5

## Data Availability

All data needed to evaluate the conclusions in the paper are present in the paper or the Supplementary Information. Additional data related to this paper may be requested from the corresponding authors. Source data are provided with this paper.

## References

[1] Kim, I. H. et al. Human-muscle-inspired single fibre actuator with reversible percolation. *Nat. Nanotechnol.* **17**, 1198–1205 (2022). 36302962 10.1038/s41565-022-01220-2 PMC9646516

[2] Zhang, X. A. et al. Dynamic gating of infrared radiation in a textile. *Science* **363**, 619–623 (2019). 30733415 10.1126/science.aau1217

[3] Montero de Espinosa, L., Meesorn, W., Moatsou, D. & Weder, C. Bioinspired polymer systems with stimuli-responsive mechanical properties. *Chem. Rev.* **117**, 12851–12892 (2017). 28752995 10.1021/acs.chemrev.7b00168

[4] Tonazzini, A. et al. Variable Stiffness Fiber with Self-Healing Capability. *Adv. Mater.* **28**, 10142–10148 (2016). 27689347 10.1002/adma.201602580

[5] Tao, X. *Handbook of Smart Textiles* (Springer, 2015).

[6] Haines, C. S. et al. Artificial muscles from fishing line and sewing thread. *Science* **343**, 868–872 (2014). 24558156 10.1126/science.1246906

[7] Zeng, W. et al. Fiber-based wearable electronics: a review of materials, fabrication, devices, and applications. *Adv. Mater.* **26**, 5310–5336 (2014). 24943999 10.1002/adma.201400633

[8] Chortos, A. et al. Printing reconfigurable bundles of dielectric elastomer fibers. *Adv. Funct. Mater.* **31**, 2010643 (2021).

[9] Chu, H. et al. Unipolar stroke, electroosmotic pump carbon nanotube yarn muscles. *Science* **371**, 494–498 (2021). 33510023 10.1126/science.abc4538

[10] Leng, J., Lan, X., Liu, Y. & Du, S. Shape-memory polymers and their composites: stimulus methods and applications. *Prog. Mater Sci.* **56**, 1077–1135 (2011).

[11] Li, M., Pal, A., Aghakhani, A., Pena-Francesch, A. & Sitti, M. Soft actuators for real-world applications. *Nat. Rev. Mater.* **7**, 235–249 (2022). 35474944 10.1038/s41578-021-00389-7 PMC7612659

[12] Xue, E., Liu, L., Wu, W. & Wang, B. Soft fiber/textile actuators: from design strategies to diverse applications. *ACS Nano* **18**, 89–118 (2024). 38146868 10.1021/acsnano.3c09307

[13] Sanchez, V., Walsh, C. J. & Wood, R. J. Textile technology for soft robotic and autonomous garments. *Adv. Funct. Mater.* **31**, 2008278 (2021).

[14] Xiong, J., Chen, J. & Lee, P. S. Functional fibers and fabrics for soft robotics, wearables, and human–robot interface. *Adv. Mater.* **33**, 2002640 (2021). 33025662 10.1002/adma.202002640 PMC11468729

[15] Chen, T., Pauly, M. & Reis, P. M. A reprogrammable mechanical metamaterial with stable memory. *Nature* **589**, 386–390 (2021). 33473228 10.1038/s41586-020-03123-5

[16] Yun, G. et al. Liquid metal-filled magnetorheological elastomer with positive piezoconductivity. *Nat. Commun.* **10**, 1300 (2019). 30899009 10.1038/s41467-019-09325-4 PMC6428896

[17] Li, R. & Sun, L. Z. Dynamic mechanical behavior of magnetorheological nanocomposites filled with carbon nanotubes. *Appl. Phys. Lett.* **99**, 131912–131912 (2011).

[18] Wereley, N. M. (ed.) *Magnetorheology: Advances and Applications* (The Royal Society of Chemistry, 2013).

[19] Carlson, J. D. & Jolly, M. R. MR fluid, foam and elastomer devices. *Mechatronics* **10**, 555–569 (2000).

[20] Kim, Y. & Zhao, X. Magnetic soft materials and robots. *Chem. Rev.* **122**, 5317–5364 (2022). 35104403 10.1021/acs.chemrev.1c00481 PMC9211764

[21] Mark, R. J., Carlson, J. D. & Beth, C. M. A model of the behaviour of magnetorheological materials. *Smart Mater. Struct.* **5**, 607–614 (1996).

[22] Bastola, A. K. & Hossain, M. A review on magneto-mechanical characterizations of magnetorheological elastomers. *Compos. B Eng.* **200**, 108348 (2020).

[23] Kim, J. et al. Programming magnetic anisotropy in polymeric microactuators. *Nat. Mater.* **10**, 747–752 (2011). 21822261 10.1038/nmat3090

[24] Xuan, S., Xu, Y., Liu, T. & Gong, X. Recent progress on the magnetorheological plastomers. *Int. J. Smart Nano Mater.* **6**, 135–148 (2015).

[25] Li, Y., Li, J., Li, W. & Du, H. A state-of-the-art review on magnetorheological elastomer devices. *Smart Mater. Struct.* **23**, 123001 (2014).

[26] International Commission on Non-Ionizing Radiation Protection. Guidelines on limits of exposure to static magnetic fields. *Health Phys.* **96**, 504–514 (2009).19276710

[27] Lee, Y. et al. Magnetically actuated fiber-based soft robots. *Adv. Mater.* **35**, 2301916 (2023). 10.1002/adma.202301916 PMC10526629 37269476

[28] Fan, J. et al. Magnetic fiber robots with multiscale functional structures at the distal end. *Adv. Funct. Mater.* **34**, 2309424 (2024).

[29] Kim, Y., Parada, G. A., Liu, S. & Zhao, X. Ferromagnetic soft continuum robots. *Sci. Robot.* **4**, eaax7329 (2019). 33137788 10.1126/scirobotics.aax7329

[30] Zhao, X. et al. Soft fibers with magnetoelasticity for wearable electronics. *Nat. Commun.* **12**, 6755 (2021). 34799591 10.1038/s41467-021-27066-1 PMC8604991

[31] Zhang, Y. et al. Coaxially printed magnetic mechanical electrical hybrid structures with actuation and sensing functionalities. *Nat. Commun.* **14**, 4428 (2023). 37481621 10.1038/s41467-023-40109-z PMC10363174

[32] Cooper, C. B. et al. Autonomous alignment and healing in multilayer soft electronics using immiscible dynamic polymers. *Science* **380**, 935–941 (2023). 37262169 10.1126/science.adh0619

[33] Huang, Y. et al. Magnetic-assisted, self-healable, yarn-based supercapacitor. *ACS Nano* **9**, 6242–6251 (2015). 26029976 10.1021/acsnano.5b01602

[34] Banerjee, H. et al. Soft multimaterial magnetic fibers and textiles. *Adv. Mater.* **35**, 2212202 (2023). 10.1002/adma.202212202 37080546

[35] Ji, X., Xu, Y., Zhang, W., Cui, L. & Liu, J. Review of functionalization, structure and properties of graphene/polymer composite fibers. *Compos. A Appl. Sci Manuf.* **87**, 29–45 (2016).

[36] Luo, C. J., Stoyanov, S. D., Stride, E., Pelan, E. & Edirisinghe, M. Electrospinning versus fibre production methods: from specifics to technological convergence. *Chem. Soc. Rev.* **41**, 4708–4735 (2012). 22618026 10.1039/c2cs35083a

[37] Chien, A.-T. et al. High-strength superparamagnetic composite fibers. *Polymer* **55**, 4116–4124 (2014).

[38] Ahn, B. W. & Kang, T. J. Preparation and characterization of magnetic nanofibers with iron oxide nanoparticles and poly(ethylene terephthalate). *J. Appl. Polym. Sci.* **125**, 1567–1575 (2012).

[39] Zhang, H. et al. Magnetic nanoparticle-loaded electrospun polymeric nanofibers for tissue engineering. *Mater. Sci. Eng. C* **73**, 537–543 (2017). 10.1016/j.msec.2016.12.116 28183642

[40] Schrödner, M. & Pflug, G. Magnetomechanical properties of composites and fibers made from thermoplastic elastomers (TPE) and carbonyl iron powder (CIP). *J. Magn. Magn. Mater.* **454**, 258–263 (2018).

[41] Farshad, M., Clemens, F. & Le Roux, M. Magnetoactive polymer composite fibers and fabrics - processing and mechanical characterization. *J. Thermoplast. Compos. Mater.* **20**, 65–74 (2007).

[42] Schmauch, M. M., Mishra, S. R., Evans, B. A., Velev, O. D. & Tracy, J. B. Chained iron microparticles for directionally controlled actuation of soft robots. *ACS Appl. Mater. Interfaces* **9**, 11895–11901 (2017). 28349697 10.1021/acsami.7b01209

[43] Guo, Y. et al. Achieving high stiffness range of force feedback gloves using variable stiffness mechanism. In *Proc.**2019 IEEE World Haptics Conference* 205–210 (IEEE, 2019).

[44] Friston, S., Griffith, E., Swapp, D., Marshall, A. & Steed, A. position-based control of under-constrained haptics: a system for the Dexmo glove. *IEEE Robot. Autom. Lett.* **4**, 3497–3504 (2019).

[45] Yin, J., Hinchet, R., Shea, H. & Majidi, C. Wearable soft technologies for haptic sensing and feedback. *Adv. Funct. Mater.* **31**, 2007428 (2021).

[46] Peacock, A. J. & Calhoun, A. in *Polymer Chemistry* 267–283 (Hanser, 2006).

[47] Xia, X., Cai, S. & Xie, C. Preparation, structure and thermal stability of Cu/LDPE nanocomposites. *Mater. Chem. Phys.* **95**, 122–129 (2006).

[48] Gao, Q.-F., Hattab, M., Jrad, M., Fleureau, J.-M. & Hicher, P.-Y. Microstructural organization of remoulded clays in relation with dilatancy/contractancy phenomena. *Acta Geotech.* **15**, 223–243 (2020).

