# Supplementary material for: Vector-stimuli-responsive magnetorheological fibrous materials
Source: Nature. 2025 Nov 5;647(8089):375–82. doi: 10.1038/s41586-025-09706-4 (PMC12611776; doi:10.1038/s41586-025-09706-4)
Supplement: Supplementary file 1 — This file contains Supplementary Notes 1–10, Supplementary Figs. 1–30, Supplementary Tables 1 and 2, and references. [file 41586_2025_9706_MOESM1_ESM.pdf]

nature portfolio

https://doi.org/10.1038/s41586-025-09706-4

Supplementary information

Vector-stimuli-responsive magnetorheological fibrous materials

In the format provided by the authors and unedited

Supplementary information for

Vector-stimuli-responsive magnetorheological fibrous materials

Junhong Pu*, Haiqiong Li, Jin Liu, Ke Li, and Xiaoming Tao*

*Correspondence: xiao-ming.tao@polyu.edu.hk; Junhong.pu@polyu.edu.hk

Supplementary information includes:

Supplementary Note 1-10

Supplementary Figure 1-30

Supplementary Table 1-2

References

Supplementary Note

Supplementary Note 1

Model of MR yarn bending actuation and stiffening

For MR thin rod consisting of polymer and soft-magnetic particles, an energy approach is employed. Considering the mechanical energy of a bent thin rod, the magnetic energy due to the particle magnetization within an applied uniform magnetic field and additional demagnetizing effects caused by anisotropic magnetization of the rod, the total energy U of the magnetic rod is their sum its elastic energy U_e and magnetic energy U_m:

$$U = U_e + U_m \qquad (1)$$

Assuming that the thin rod behaves as a classic linear elastic anisotropic beam, its axial stretching and twisting can be ignored compared with bending, then the elastic energy stored in the bent rod is therefore just that contributed by bending deformation, which can calculate using the formula:

$$U_e = \frac{EI}{2}\int_0^l \left(\frac{d^2\delta}{ds^2}\right)^2 ds \qquad (2)$$

where E is the Young's modulus, I is the second moment of inertia, δ is the deflection, s is the position along the rod length l.

By applying a uniform magnetic field, the magnetizable rod is magnetized with a magnetization of $\boldsymbol{M}$, which in turn leading to the demagnetizing field $\boldsymbol{H_m}$. The magnetostatic energy of the rod body is defined by[1]

$$U_m = -\frac{\mu_0 A}{2}\int_0^l \boldsymbol{H_m}\cdot\boldsymbol{M}ds \qquad (3)$$

where A is the area of the rod cross-section, μ_0 is the permeability of free space.

- *Bending actuation*

In this scenario, the magnetic field is applied in a direction deviating from the axis of a magnetic rod with an angle ψ, its one end is fixed at point O on the horizontal shaded surface (Supplementary Fig. 1). Since the rod containing soft-magnetic particles has negligible remanence magnetization, any magnetic moment is the result of shape anisotropy due to the demagnetizing field along its radial axes. This causes its tendency to align with the applied field with an angle θ. The magnetic energy is minimized when the magnetization of the cantilever lies along its long axis. The elastic energy of the bent rod therefore increases with a higher curvature.

Considering the MR yarn is composed of six helical fibers wrapped around a center straight fiber following the yarn axis, it presents a cross-sectional symmetry similar to that of a cylinder, with a diameter three times that of an individual fiber. Additionally, the heat-setting process alleviates residual stress within each twisted fiber, and eliminates the majority of internal stress and friction inside the yarn. Hence, for analytical investigation, the long and thin yarn can be effectively modeled as a cylindrical rod.

To relieves much of the complexity of in equation (3) stemming from the fact that shape anisotropy affects both the direction and magnitude of the magnetization $\boldsymbol{M}$. The simplification can be made that the magnitude of $\boldsymbol{M}$ is dependent only on the applied field $\boldsymbol{H}$ and the initial orientation of the rod[2,3]. In the case of thin rod with a high aspect ratio over 10, the demagnetization factors parallel and perpendicular to the long axis is approximated to 0 and 0.5, respectively[4]. Thus, the magnetostatic energy can be approximately calculated by[5],

$$U_m = \frac{\mu_0 Al}{2} \frac{{\chi_m}^2}{\chi_m+2} |\boldsymbol{H}|^2 \sin^2\theta \qquad (4)$$

where χ_m denotes the volume magnetic susceptibility of the composite materials. θ is the angle between the directions of the magnetic field and the rod axis.

Assume the rod bends into a part of a circle with radius of R, we have $\frac{d^2x}{dy^2} = \frac{1}{R} = \frac{2\varphi}{l}$. Refer to equation (1), (2), and (4), the total energy of the bent rod can be expressed as:

$$U = \frac{EIl}{2}\left(\frac{2\varphi}{l}\right)^2 - \frac{\mu_0 Al}{2} \frac{{\chi_m}^2}{\chi_m+2} |\boldsymbol{H}|^2 \sin^2(\psi - \varphi) \qquad (5)$$

Taking a derivative of φ in equation (5) and make it equal to zero

$$0 = \frac{4EI\varphi}{l} - \mu_0 Al \frac{{\chi_m}^2}{\chi_m+2} |\boldsymbol{H}|^2 \sin(\psi - \varphi)\cos(\psi - \varphi) \qquad (6)$$

The magnetic moment causes the rod to bend until the mechanical moment of the bending elastic rod balance out. The bending angle of equilibrium state achieved with required magnetization is given by

$$\varphi = \frac{\mu_0}{8} |\boldsymbol{H}|^2 \frac{{\chi_m}^2}{(\chi_m+2)E} \frac{Al^2}{I} \sin(2\psi - 2\varphi) \qquad (7)$$

The expression indicates that the magnetic moment is maximized when θ = 45°. Assume the field is applied in this orientational angle. Given CIPs are typical soft-magnetic materials with high $\chi_m \gg 1$, the above equation can be further simplified as:

$$\varphi = \frac{\mu_0}{8} |\boldsymbol{H}|^2 \frac{\chi_m}{E} \frac{Al^2}{I} \qquad (8)$$

The second part, $|\boldsymbol{H}|^2$ means the bending angle is proportional to the square of the magnetic field strength, however it tends to be saturated after a certain threshold point. The third part, $\frac{\chi_m}{E}$ of this expression contains only material properties, reflecting that the materials with a high susceptibility and a low modulus are preferred for magnetic bending actuation. The fourth part, $\frac{Al^2}{I}$ contains all of the geometrical parameters of the rod. More precisely, $\frac{A}{I}$ is considered with respect to the geometrical design of yarn since the total length can be easily adjusted.

In our cases, we adopt the idealized helical yarn geometry for theoretical studies on the yarn-structured rod. It has the same length and apparent diameter as the cylindrical rod. That is to say, the radius of each fiber is $\frac{r}{3}$. For the geometrical term, the cross-section area of the MR yarn A_y can be expressed as,

$$A_y = \frac{7}{9}\pi r^2 \qquad (9)$$

The second moment of inertia I_y of the MR yarn model as six helical fibers wrapped around a center straight fiber ignoring the internal friction between fibers can be calculated as[6],

$$I_y = \frac{\pi}{4}\left(\frac{r}{3}\right)^4 \left(1 + \frac{12\cos\alpha}{2+\nu\sin^2\alpha}\right) \qquad (10)$$

where α is the surface helical angle of the yarn, ν is the Poisson ratio of fiber. Integrating equations (9) and (10) we obtain the geometrical indicator, $\frac{A_y}{I_y}$, for yarn as,

$$\frac{A_y}{I_y} = \frac{252}{r^2}\left(1 + \frac{12\cos\alpha}{2+\nu\sin^2\alpha}\right)^{-1} \qquad (11)$$

It indicates a smaller fiber diameter and higher degree of twist leading to larger $\frac{A_y}{I_y}$.

- *Stiffening*

In this situation, we consider the simply supported rod in a uniform magnetic field. The rod is subjected to a downward load $\boldsymbol{F}$ at the center, causing it to bend (Supplementary Fig. 2). The elastic energy is stored in the bending rod. Simultaneously, the magnetic field induces magnetization of the rod leading to the increase of magnetic energy of the rod after deflection. Thus, the rod stiffens up as the magnetic field strength becomes larger. At equilibrium, the work W done by the load is equal to the sum of the elastic and magnetic energy increase in the rod, ref to (1), we have

$$W = U_e + U_m \qquad (12)$$

Noted by the symmetry, we study the right half of the rod that can be regarded as a rod cantilevered at point O. With the similar simplification in the magnetization of the rod in the first case. For the right half rod, the energies are expressed as,

$$\frac{E_a I}{2}\int_0^{\frac{l}{2}}\left(\frac{d^2y}{dx^2}\right)^2 dx = \frac{EI}{2}\int_0^{\frac{l}{2}}\left(\frac{d^2y}{dx^2}\right)^2 dx + \frac{\mu_0 A\frac{l}{2}}{2}\frac{{\chi_m}^2}{\chi_m+2}|\boldsymbol{H}|^2\sin^2(\varphi) \qquad (13)$$

where E_a is the apparent modulus of the rod. With the same approximation on the shape of the right half rod with length of $\frac{l}{2}$ bent into a part of arc with radius of R, here we get $\frac{d^2y}{dx^2} = \frac{1}{R}$ and $\sin(\varphi) = \frac{l}{4R}$, the above equation is processed into,

$$\frac{E_a I\frac{l}{2}}{2}\left(\frac{1}{R}\right)^2 = \frac{EI\frac{l}{2}}{2}\left(\frac{1}{R}\right)^2 + \frac{\mu_0 A\frac{l}{2}}{2}\frac{{\chi_m}^2}{\chi_m+2}|\boldsymbol{H}|^2\left(\frac{l}{4R}\right)^2 \qquad (14)$$

Rearranging the equation to solve for the relative change of apparent bending rigidity $(E_a I - EI)/EI$ gives:

$$\frac{E_a I - EI}{EI} = \frac{\mu_0}{16}|\boldsymbol{H}|^2\frac{{\chi_m}^2}{(\chi_m+2)E}\frac{Al^2}{I} \qquad (15)$$

Given CIPs are typical soft-magnetic material with high $\chi_m \gg 1$, the above equation can be simplified as:

$$\frac{E_a I - EI}{EI} = \frac{\mu_0}{16}|\boldsymbol{H}|^2\frac{\chi_m}{E}\frac{Al^2}{I} \qquad (16)$$

The right part of the equation is similar to the right part of (8), thus the same material and geometrical design as the bending actuator can result in a broader range of apparent bending rigidity controlled by magnetic fields.

<u>Influencing factors in MR yarn bending actuation and stiffening</u>

The discussion was based on the analytic models of MR yarn bending and stiffening. The bending actuation angle and bending rigidity were calculated with equation (7) and (16), respectively. $\frac{A_y}{I_y}$ was calculated with equation (11).

The differences between parallel and perpendicular magnetization of the MR yarn were within 40% across the spectrum (Fig. 2i). The magnetic fields were applied nearly perpendicular to the cantilevered MR yarn for bending actuation, and parallel to the simple supported MR yarn for stiffening. We employed the fitted perpendicular and parallel magnetization curves to describe the magnetization of MR yarn ($|\boldsymbol{M}| = \chi_m|\boldsymbol{H}|$) in any position during bending and

stiffening, respectively (Supplementary Fig. 9). The magnetic field strength in the air was calculated with $|\boldsymbol{B}| = \mu_0|\boldsymbol{H}|$. Related parameters were all acquired experimentally.

<u>Magnetization properties of MR yarn in axial and radial directions</u>

As seen in Supplementary Fig. 9, at the same magnetic field strength, the magnetization is higher when the applied field is parallel than perpendicular to the yarn axis, similar to the behavior of an MR cylinder with a high aspect ratio. This creates easy and hard axes within the yarn.

The MR yarn consists of CIPs is a typical ferromagnetic material, whose magnetization spectra can be described by the Langevin model[7]

$$|\boldsymbol{M}| = \left[\coth\left(\frac{m\mu_0|\boldsymbol{H}|}{kT}\right) - \frac{kT}{m\mu_0|\boldsymbol{H}|}\right]|\boldsymbol{M}^*| \qquad (17)$$

where m is the absolute value of magnetic moment, k is the Boltzmann constant, T is the temperature, and $|\boldsymbol{M}^*|$ is the saturation magnetization. The experimental results of the magnetization of the yarn in two directions are well described with the fitted curves, which are used for the prediction of actuation performance.

Supplementary Note 2

The spinnability of a polymer is related to the ability of molten polymer strands to be drawn without breaking. In particular, the viscous behavior dominates the shear and elongational flow at a comparatively low velocity near the spinneret, while the elastic part becomes more important with increasing velocity away from the spinneret.

With an increase in CIP content to 70 wt%, all composites exhibite a slight increase in viscosity (Extended Data Fig. 1a), which is attributed to interactions between polymer chains and CIPs. Upon further increasing the CIP content from 70 to 80 wt%, an order of enhancement in viscosity is observed, along with a more pronounced shear-thinning behavior at low frequencies. Additionally, tan δ decreases below 1 (Fig. 2b), indicating a significant solid-like behavior in the 80 wt% composite. Both observations suggest the development of an interconnected network of CIPs. Such high-viscosity and solid-like composite is difficult to extrude from the spinneret.

The elastic behavior of molten composites with filler contents of 30, 50, and 70 wt% show a similar increase in G' as compared with LDPE (Extended Data Fig. 1b). This increase is attributed to the restriction of large-scale relaxation of polymer chains due to interactions with particle surfaces. The formation of a CIP network within the 80 wt% composite lead to a significant improvement in G'. The plateau modulus (G_0), a rough indicator of spinnability, exhibits an inverse relationship with the maximum draw ratio of the spun fiber[8]. In other words, the drawing of composite fibers shows a variation in equilibrium elastic modulus that is strongly influenced by filler content. This phenomenon is associated with plastic instability, which could lead to draw instability, resulting in diameter variations and rupture of spun fibers. G_0 is extracted from G' curves when tan δ reaches its minimum[9]. With increasing filler content, G_0 of LDPE increases from 39 Pa to around 300 Pa for 30, 50, and 70 wt% composites. A

further 10% increase to 80 wt% escalates G_0 to 3892 Pa, which could result in substantial draw instability.

Supplementary Note 3

We examined the evolution of the fiber structure after spinning out of the spinneret, as depicted in Extended Data Fig. 3a. The filament diameter decreases consistently from the spinneret to the guide wheel during the drawing process (Extended Data Fig. 3b). An initial rapid thinning of the filament diameter, from 750 to 155 μm, occurs over the approximately 30 mm range just below the spinneret, corresponding to a 23-time increase in the filament length. The stretching flow field within the molten filament sufficiently orients the chains, resulting in an axial strain elongation of 41% for random chains (Fig. 2d) and radial movement of CIPs out of the unbound surface (i and ii in Extended Data Fig. 3c). Subsequently, filament thinning gradually slows down and stops at approximately 200 mm below the spinneret due to cooling and solidification of the molten filament. During this stage, stretching-induced tension further elongates the amorphous regions in the deformation direction along with intense cavitation around CIPs (iii in Extended Data Fig. 3c). Further drawing by 7.5 times, the diameter of the fiber reduces to 57 μm. The total draw ratio of 173 enables a high degree of chain orientation, which contracts by 66% after full relaxation (Fig. 2d).

Supplementary Note 4

To illustrate the attraction between wrapping fibers, we conducted an MR fiber interaction test. Considering the angle between the magnetic field and any small portion of the wrapping fibers almost equal to the surface helical angle. We placed two MR fibers, each 30 mm in length, parallel to each other at a distance of 120 μm, with both ends fixed on a horizontal plane for characterization. Magnetic fields were applied horizontally at various angles relative to the length of the MR fibers. The minimum magnetic field strength needed to bring the two parallel fibers together at specific angles was recorded. From Extended Data Fig. 5a and b, as the angle increases, the required magnetic field strength decreases rapidly, reaching its minimum at 90°. This phenomenon indicates that the mutual attraction of the magnetized fibers stems from the induced demagnetization fields of the two fibers. When a MR yarn is subjected to a magnetic field along its length direction, all wrapping MR fibers attract together through their demagnetization fields, leading to the contraction of MR yarn (Extended Data Fig. 5c and d). This process, coupled with a high frictional coefficient (0.36) between fiber surfaces, leads to higher energy dissipation when bending the magnetized MR yarn (Supplementary Fig. 10), thus significantly contributing to its increased bending rigidity.

Supplementary Note 5

Safety assessment is essential, especially given the intended use of stimuli-responsive fibrous materials in wearable applications, such as gloves and ventilated fabrics, that come into frequent, close skin contact. Safety evaluation on stimuli including voltage, temperature, and static magnetic fields included in Supplementary Table 1 that might potentially have direct biological effects on ordinary users. To ensure broad applicability, this evaluation excludes indirect hazards like secondary effects and interference with implanted medical devices as well

as clinical applications such as specific treatment and examination, as these factors are outside our purview. The evaluation draws on widely accepted standards and guidelines, considering specific parameters to create a robust yet accessible safety profile suitable for daily, non-specialist wearable use.

Electrical Safety Evaluation of Dielectric Fibers

Dielectric elastomer actuators, that operate at high voltages (>500 V), pose risks if accidentally discharged to a human. Our evaluation is based on a useful framework for evaluating DEA safety provided by C. Menon et al.[10], which is based on standards from Underwriters Laboratories and International Electrotechnical Commission that limit the safe amplitude and duration of electrical currents passing through the body. They defined a critical capacitance threshold (C_{cr}) for safe DEA operation based on theoretical and experimental validation, which the DEA capacitance must stay below at the applied voltage to avoid potential hazards in a worst-case scenario where a fully charged actuator discharges into a human body with a minimum resistance of 500 Ω.

For the fiber-based dielectric bending actuators, each produces small bending angles (0.12, 0.29, and 0.11 ° mm^{-1}) at its maximum voltage, resulting in minor deformation from their rest states. To assess electrical safety, capacitances were calculated in their unactuated state, using structural parameters and dielectric constants, and evaluated against C_{cr} at their respective voltages. The actuator of Ref. 36 bends with two fibers activated, yielding a total capacitance of 12.4 nF at 0.8 kV. This is safely below the C_{cr} of 1.6 μF. The actuator of Ref. 37 bends with three fibers selectively activated, with a total capacitance of 127.2 pF at 7 kV. This is safely below the C_{cr} of 150 nF. The tubular dielectric fiber of Ref. 38 bends at 10 kV with a capacitance of 22.4 pF, which is safely below the C_{cr} of 80 nF.

Temperature Safety Evaluation for Thermal-Responsive Fibers/Yarns

Thermal-responsive fibers and yarns can reach elevated temperatures during activation, raising safety concerns for close-to-body applications, where accidental contact with hot surfaces poses a risk of burns. Our temperature safety evaluation follows CENELEC GUIDE 29 from the European Committee for Electrotechnical Standardization, which identifies surface temperature, contact duration, and material type as key factors in burn risk. Given that these fibers are primarily polymer-based, we use burn threshold criteria specific to plastics to ensure accuracy.

Operating temperatures above 90.5 ℃ exceed the safe threshold and are considered unsafe for any contact. For temperatures below this threshold, we determine the maximum safe contact time, named time limit, using the burn threshold curve, where longer time limit indicate a higher safety margin. Overall, the most thermal-responsive fibers and yarns provide only a few seconds of safe contact time. For example, SMP yarn reaches a maximum temperature of 83 ℃, allowing safe contact for up to 1.5 seconds. For the fiber operating at 60 ℃, it permits up to 60 seconds of safe contact, which may still not satisfy wearable applications requiring continuous use over several minutes.

Magnetic Safety Evaluation of Magnetic Fibers

Flexible magnetic fibers containing magnetic fillers are actuated through the application of a static or low-frequency magnetic field with controllable direction and strength. For close-to-

body applications, human body is also exposed to magnetic fields, warranting evaluation of potential biological effects. Our safety assessment aligns with the International Commission on Non-Ionizing Radiation Protection (ICNIRP) guidelines, which set a public exposure limit of 400 mT for magnetic flux density on any body part.

Overall, this analysis suggests that all the magnetic fibrous materials, including our MR yarn, with known field strengths operate safely within the ICNIRP limit, confirming their safety for wearable applications. An exception is the certain fiber of Ref. 38 with unreported magnetic field strength, where safety cannot be verified.

Supplementary Note 6

We conducted significance tests using Spearman's correlation coefficient and calculated p-values, with a significance level (α) set at 0.05. The null hypothesis states that the correlation between float length and moment density is not significant. For a given magnetic field strength, nine samples were tested: three float lengths (1, 2, and 4) with practical measured lengths of yarn float of 3.8, 5.3, and 7.3 mm, respectively, each with three replicates. These replicates were used to calculate Spearman's correlation coefficient and corresponding p-values. The results are illustrated in the five heatmaps (Supplementary Fig. 12). Using a one-tailed test and setting the critical value for Spearman's coefficient at 0.6 (based on an alpha of 0.05), we found that all calculated coefficients exceeded 0.6, and all p-values were below 0.05, except in the case of the 70 mT magnetic field. This indicates that for magnetic field strengths between 140 and 300 mT, we can reject the null hypothesis, suggesting a statistically significant correlation between float length and moment density. The exception at 70 mT might due to the lower magnetic field strength, which induces a smaller moment density, potentially leading to higher relative measurement errors.

Supplementary Note 7

Under square wave magnetic fields at frequencies of 0.1-10 Hz with a peak strength of 135 mT, the yarns and fabrics retained at least 30% of their initial bending degree at 10 Hz (Extended Data Fig. 6a). The actuation response and settling times, extracted from the bending angle over time in response to magnetic actuation (Extended Data Fig. 6c), revealed response times of 0.12 s, 0.07 s, 0.22 s, and 0.17 s to reach 63.2% of the steady-state bending angles, and settling times within ±2.5% of steady-state bending angles at 0.62 s, 0.58 s, 0.53 s, and 0.52 s for the yarn, plain-weave fabric, twill-weave fabric, and satin-weave fabric, respectively.

For the cut-pile MR fabric with MR yarns (4 mm in length) packed vertically to the fabric plane, square wave magnetic fields at frequencies of 0.1-10 Hz with a peak strength of 247 mT were applied at 45° to the fabric plane. The in-plane displacement remained above 2 mm from 0.1 to 2 Hz, and then decreases to nearly 0 mm at 8 Hz (Extended Data Fig. 6b). The cut-pile fabric displayed a response time of 0.23 s and a settling time of 1.87 s (Extended Data Fig. 6c). Its dynamic performance is lower than that of the yarn and woven fabrics, primarily due to friction that resists relative movement among the densely packed MR yarns during actuation and recovery.

Supplementary Note 8

Upon the addition of CIPs, a decrease occurs in both the melting temperature (T_m) and crystallinity degree (χ) of the composite, reducing from 104 ℃ and 27.83% of LDPE to 102.83 ℃ and 27.14% of the composites, respectively (Supplementary Fig. 25a). This reduction indicates hindered polymer chain reorganization into a crystalline lattice and suppressed formation of intact spherulites[11]. Furthermore, a notable increase in the glass transition temperature (T_g) from -133.8 to -130.5 ℃ implies higher activation energy of the chain segments and restricted segmental mobility of polymer chains in the composite[11]. The lower tan δ of the composite compared to LDPE suggests strong interfacial interactions between the matrix and particles (Supplementary Fig. 25b), leading to reduced macromolecular mobility near the filler surface. The results above indicate a significant interaction between CIPs and LDPE.

The DSC reveals the presence of oriented chains in the fully drawn melt spinning fibers. As the composite is melt spun and drawn into a fiber, the onset temperature (T_{mo}) increases from 92.04 to 96.93 °C and T_m increases from 102.83 to 104.17 °C. This increase corresponds to the formation of highly oriented extended-chain crystals with a more complete structure and enhanced thermal stability compared to folded-chain crystals[12]. In addition, χ decreases from 27.14% to 22.92%. This is mainly due to the rapid cooling during the fiber spinning process, which gives insufficient time for the polymer chains to arrange themselves into crystalline structures. Thus, most of the molecular chains exist in an amorphous orientation.

As seen in the XRD scans (Supplementary Fig. 25c), LDPE shows two prominent peaks at $2\theta = 21.4°$ and 23.8°, corresponding to the (110) and (200) planes of the orthorhombic crystal structure, and a broad peak at $2\theta = 20.6°$ appearing as a halo, indicating the amorphous phase of LDPE[13,14]. For the composite and fiber with 70 wt% CIPs, peaks at $2\theta = 45°$ and 65° correspond to α-Fe from the CIPs[15]. However, the LDPE peaks are significantly attenuated in the composite and fiber due to the low polymer crystal content, which produces a much weaker signal relative to the strong filler peaks. This effect has been similarly reported in polymer composites with filler loadings above 10 wt%[16-20].

Supplementary Note 9

Mobile magnetic actuation system

A mobile magnetic field generation system that allows spatial control of its magnetic activation component relative to the target object is essential for practical applications. To meet this need, we developed a mobile magnetic actuation system comprising a pair of co-axially aligned electromagnets with opposing poles, mounted on a 6-degree-of-freedom (6-DOF) desktop robotic arm (Supplementary Fig. 17a). The magnetic field strength and size can be adjusted by controlling the electric current, pole distance, or a combination of both (Supplementary Fig. 17b-e). The robotic arm, with an extended length of 450 mm, enables precise positioning and orientation of the magnetic field. The mobile electromagnetic actuation system offers a larger workspace and greater flexibility in operation compared to the stationary desktop electromagnet.

Integrated conformable gripping device

Two cut-pile MR fabrics measuring 20×20 mm with a MR yarn height of 5 mm and density of 500 yarns cm^{-2}, were integrated onto the pole surfaces of coaxial electromagnets mounted on a parallel gripper controlled by a robotic arm. These cut-pile MR fabrics functioned as smart cushions with adjustable stiffness.

This integrated conformal gripping device provides precise control over pole distance and magnetic field strength applied to the MR fabrics. The symmetry magnetic fields in the spaces near the two poles enable equal control of the hardness of the cut-pile MR fabrics on both sides (Supplementary Fig. 18a-d). By applying a constant current of 3 A, the magnetic field strength reaches 160 and 130 mT at pole distance of 20 and 30 mm, respectively (Supplementary Fig. 18e).

The programmed handling process involved steps to ensure consistent handling of objects with varying modulus and shapes, including a worm, blueberry, tofu, potato chip, fusilli, and mung bean cake. Manipulation steps were set as follows: first, the robotic arm guided the open gripper towards the object. Then, by switching on the current at a specific intensity, we apply a magnetic field that adjusts the cushion's stiffness to match the modulus of the object. After that, the gripper closed to an extent relative to the size of object, ensuring a secure grip as the cushions conform to the object shape. Subsequently, the gripper securely held and transported the object to the target location. Upon arrival, it gently opened to release the object, followed by deactivating the magnetic field, and then returned to its starting position.

Supplementary Note 10

Remote-controllable haptic finger glove demonstration

A haptic finger glove, measuring 6 cm in length, 1.5 cm in diameter and 1.36 g in weight, was fabricated by stitching together a piece of cut-pile MR fabric with a height of 3 mm at the fingertip pad area and a plain-weave MR fabric covering the rest parts. To fit fingers of various sizes without any hindrance, highly elastic Spandex was utilized as the warp for the plain-weave MR fabrics.

A soft silicone hand mannequin with a modulus and shape similar to a human hand was utilized. The index finger is 8 cm in length (L_{finger}). A flexible pressure sensor (A201, Flexiforce) with a sensing area diameter of 9.53 mm and a thickness of 0.2 mm was integrated onto the fingertip region of the index finger. The finger glove was worn on the integrated silicone finger to facilitate tactile feedback presentation.

Kinesthetic feedback was induced by applying magnetic fields at angles λ away from the axis of the silicone finger (Supplementary Fig. 19). The finger glove generated a moment on the finger to align it with the magnetic field direction. The force (F_{ft}) in the direction along the magnetic field at the fingertip was recorded while changing the λ or current intensity. The relative force reduction (ΔF_{ft}) before and after magnetic field application related to the moment (M_{mj}) around the metacarpophalangeal joint of the finger was calculated as $M_{mj} = \Delta F_{ft} L_{finger} \sin \lambda$. Diverse kinesthetic effects can be generated by controlling rotation range and speed of end-effector and varying the amplitude, frequency, and waveform of current (Supplementary Fig. 20).

Tactile feedback was achieved by activating the cut-pile MR fabric covering the entire fingertip region (Supplementary Fig. 21). A preload was applied to the index finger, causing the fingertip region to press against a flat acrylic sheet positioned perpendicularly between two poles of the electromagnet. Electric currents at frequencies of 0.2, 0.5, and 1 Hz were applied in triangular and square waveforms, with a peak strength of 2 and 3 A. Real-time actuating force on the fingertip area was measured by the flexible pressure sensor, wirelessly transmitted to a computer, and recorded. Different tactile feedback can be achieved by modulating the amplitude, frequency, and waveform of current (Supplementary Fig. 22).

Artificial emulation of fabric handle can be achieved by this finger device since the hardness and surface roughness of the finger fabric is now regulated by the applied magnetic field. When this finger device touches the skin of human hand, the physical interface signals arising from the touch between the human skin and the fabric will be received by bioreceptors in the skin and yield sensory reactions via neural system of human, such as sensation of softness/hardness and smooth/rough. These sensations form parts of subjective fabric handle assessment by people.

Supplementary Figure

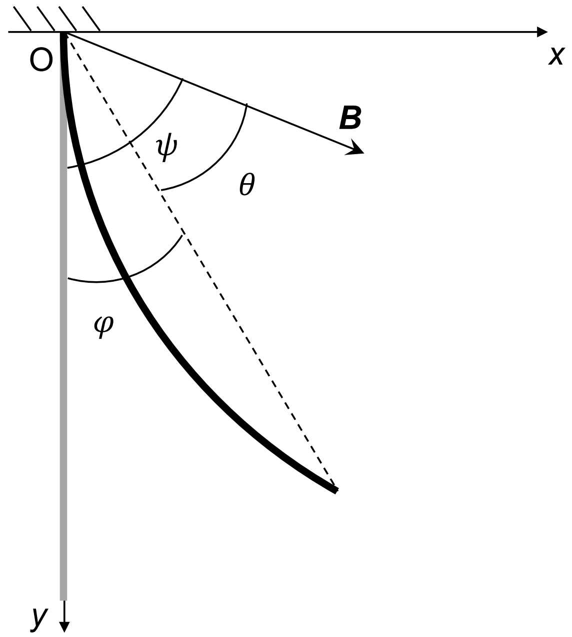

Supplementary Fig. 1. Bending model of MR yarn. A cantilevered elastic magnetic rod undergoes deflection by an angle of φ in response to the influence of a magnetic field $\boldsymbol{B}$. The gray straight line denotes the initial position, while the black curved line denotes the equilibrium position of the rod during bending actuation. ψ and θ are the angles between the magnetic field and yarn in equilibrium and initial positions, respectively.

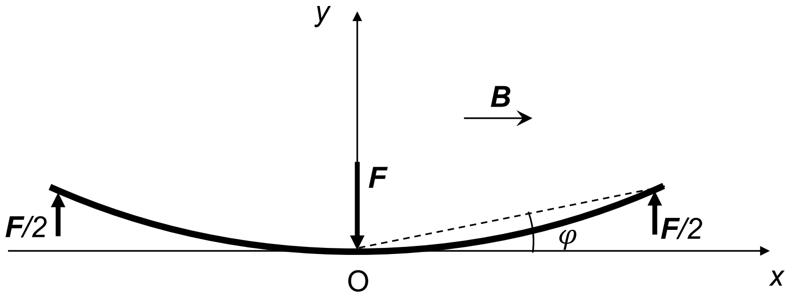

Supplementary Fig. 2. Stiffening model of MR yarn. A simply supported elastic magnetic rod experiences deflection by an angle of φ under a central vertical loading $\boldsymbol{F}$ and within a magnetic field $\boldsymbol{B}$ in the x direction.

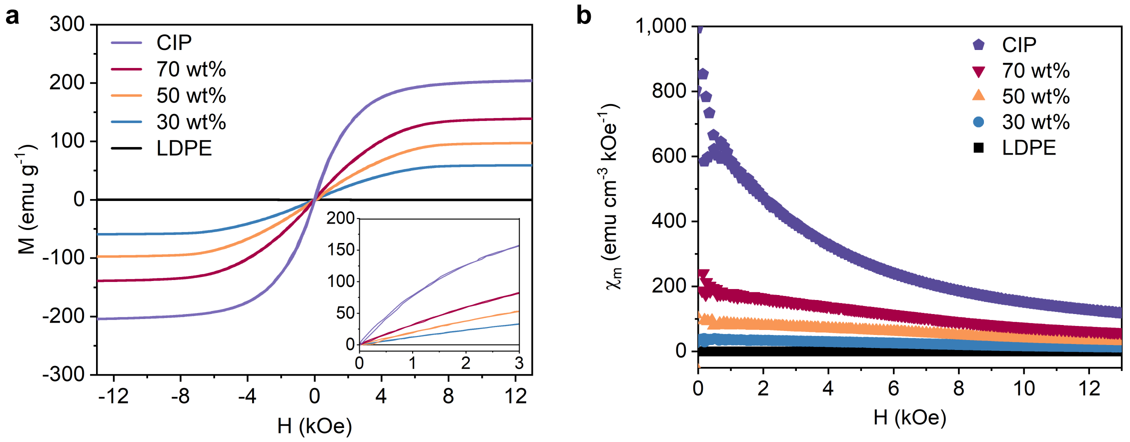

Supplementary Fig. 3. Magnetization properties of CIP, LDPE, and composites. a, Magnetization curves and **b**, Magnetic susceptibilities of CIP, LDPE, and composites.

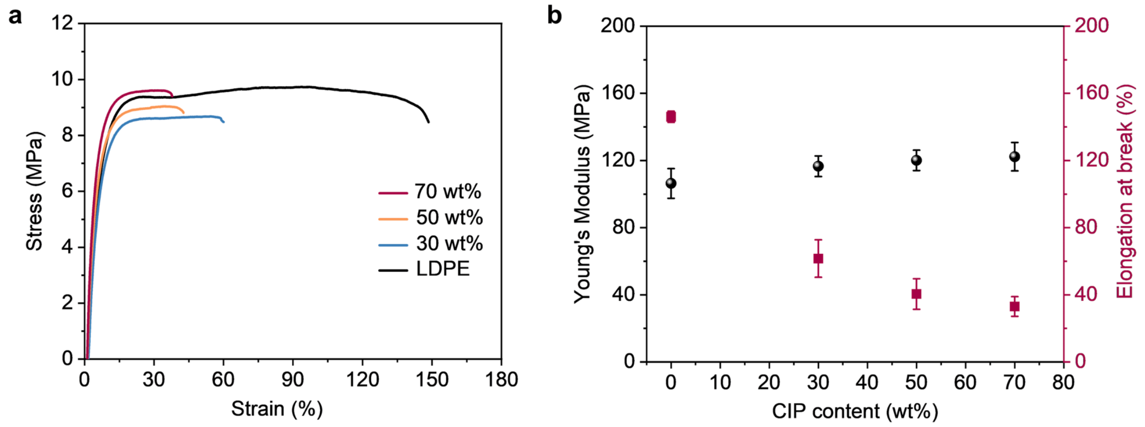

Supplementary Fig. 4. Mechanical properties of LDPE and composite bulk materials. a, Stress-strain curves and **b**, The extracted Young's modulus and elongation at break of LDPE and composite bulk materials. Error bars correspond to s.d. (n=5).

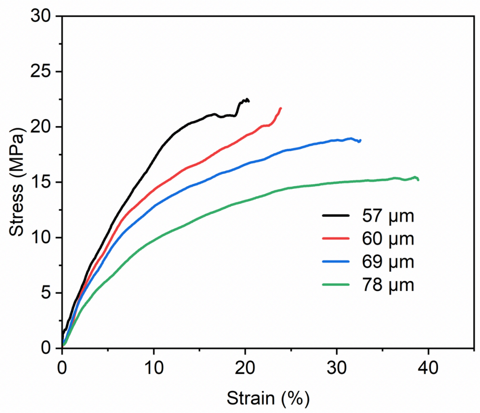

Supplementary Fig. 5. Stress-strain curves of MR fibers with different diameters.

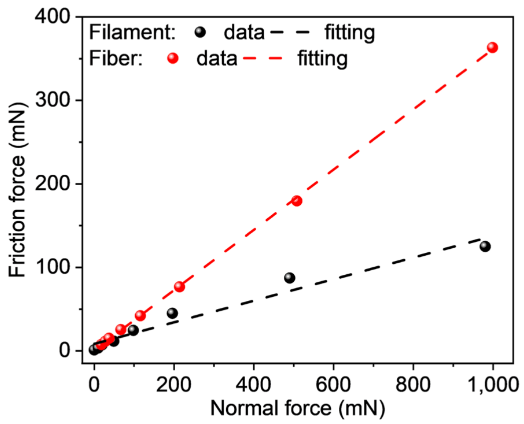

Supplementary Fig. 6. Plots of normal force against friction force for as-spun filaments and fully-drawn fibers. The static coefficients of friction between the as-spun filaments and between the fully-drawn fibers are 0.13 and 0.36, respectively.

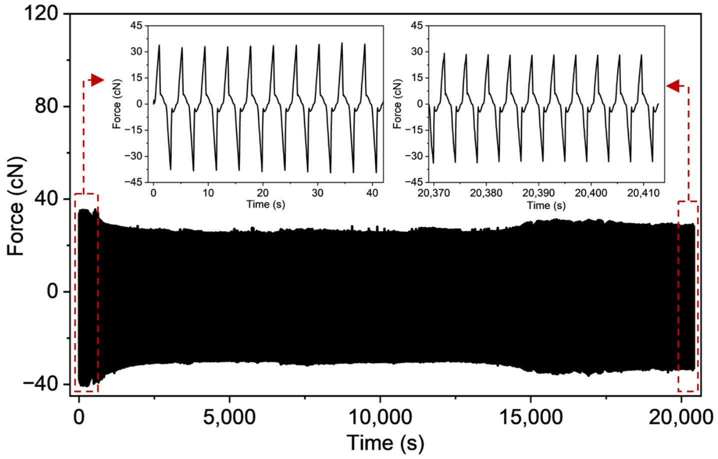

Supplementary Fig. 7. Bending durability of MR yarn. Bending fatigue test of an MR yarn subjected to around 10,000 consecutive bending cycles. Insets show the details of the first and last 10 cycles, respectively.

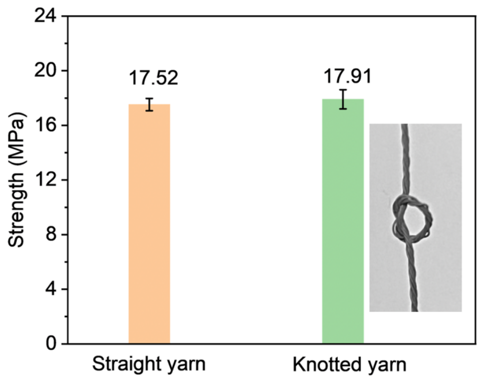

Supplementary Fig. 8. Tensile and knot strength of MR yarn. Tensile strength comparison between straight and knotted yarns. Inset photo shows the knot structure. Error bars correspond to s.d. (n=5). The knot strength is comparable to the tensile strength of the MR yarn, revealing that the yarn maintains sufficient flexibility and durability, even under the stress concentrations

introduced by knotting. Therefore, the yarn mechanical resilience suggests it can withstand typical fabric manufacturing and end-use conditions.

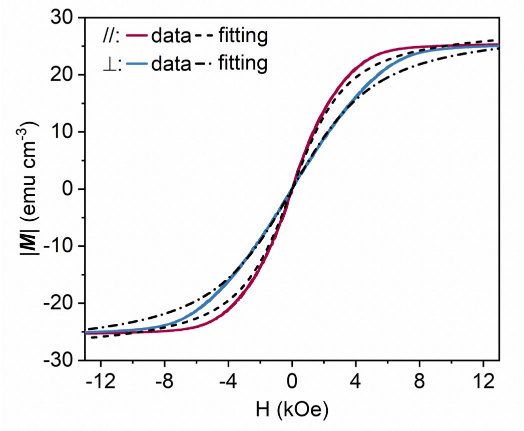

Supplementary Fig. 9. Magnetization properties of MR yarn. Measured magnetization curves of the MR yarn in the direction parallel and perpendicular to the axis, respectively. Dash lines represent the calculated curves according to the Langevin model.

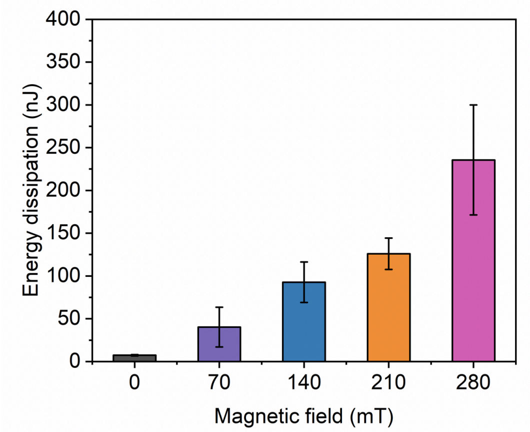

Supplementary Fig. 10. Energy dissipation of MR yarn during 3-point bending test. Energy are extracted from the hysteresis loop areas in Fig. 3e. Error bars correspond to s.d. (n=3).

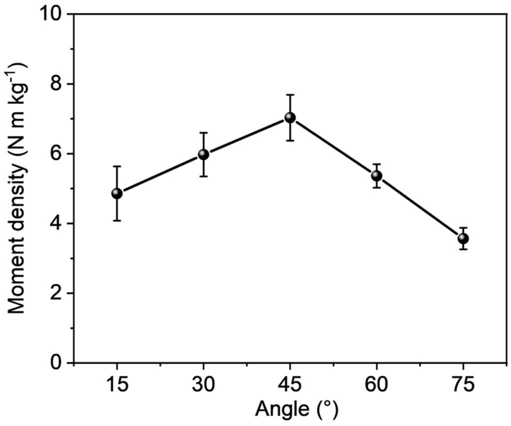

Supplementary Fig. 11. Magnetic moment density of MR yarn at various angles between the yarn axis and the magnetic field direction, with a magnetic field strength of 300 mT. Error bars correspond to s.d. (n=3).

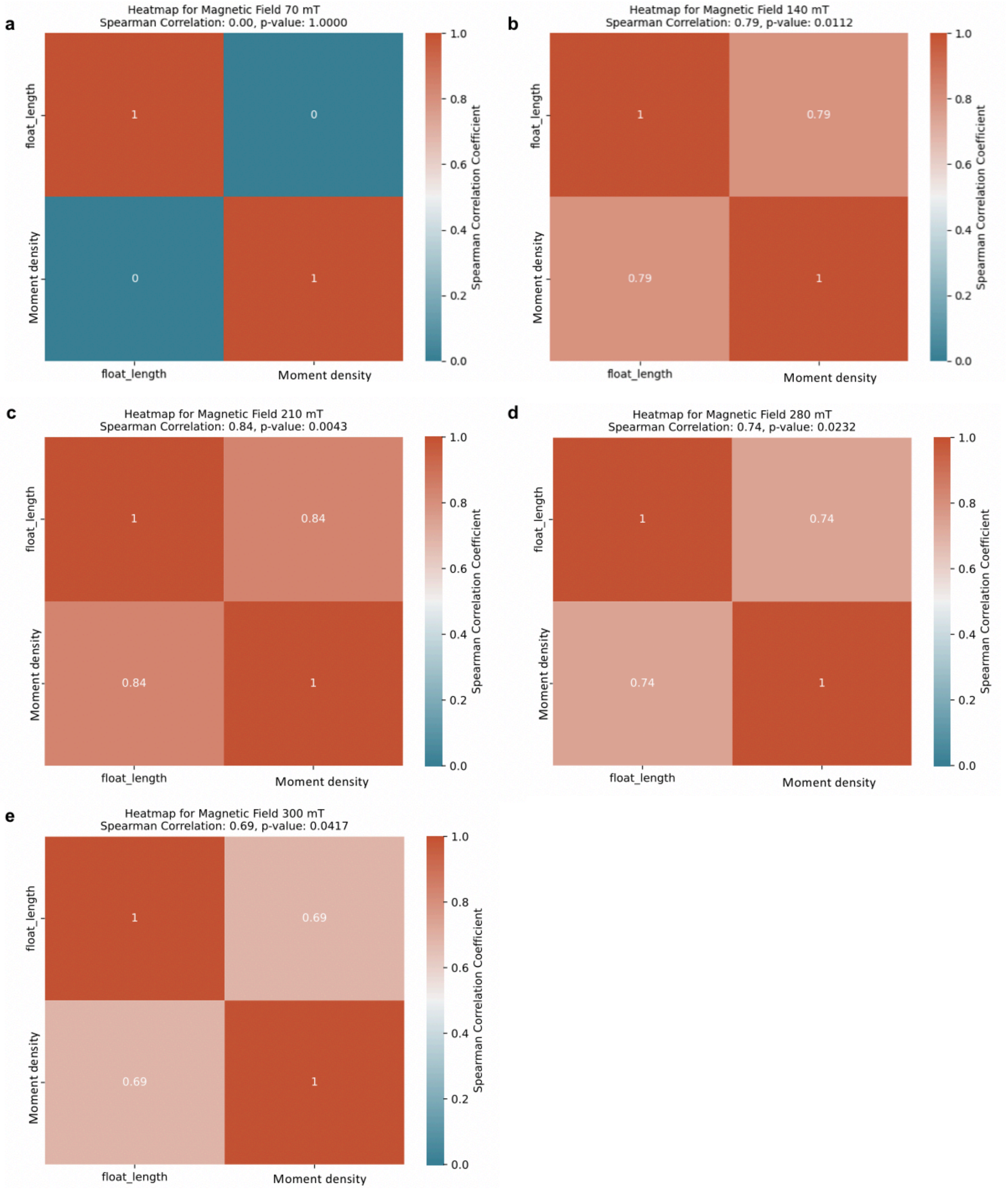

Supplementary Fig. 12. Statistical significance test. Heatmap of Spearman correlation coefficients for the relationship between float length and moment density in woven MR fabrics at magnetic field strengths of **a**, 70 mT; **b**, 140 mT; **c**, 210 mT; **d**, 280 mT; and **e**, 300 mT.

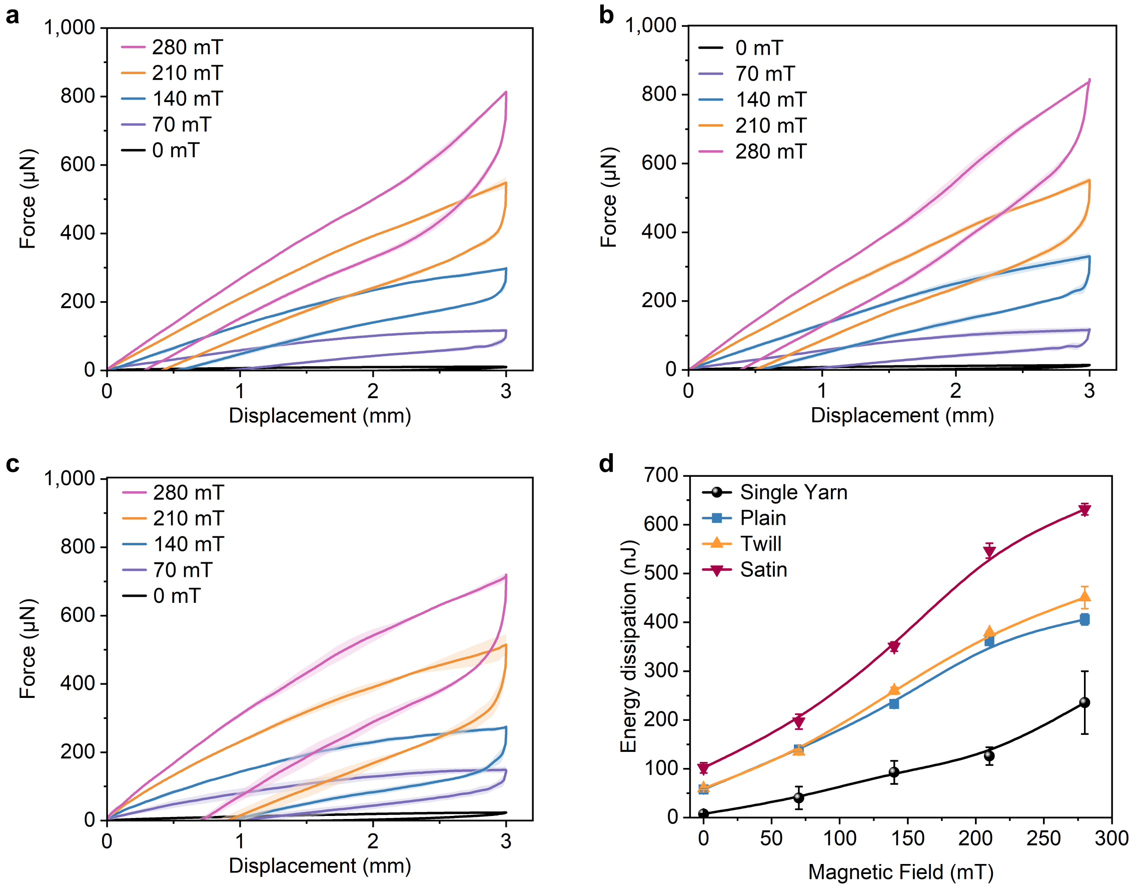

Supplementary Fig. 13. Stiffening actuation of woven MR fabrics. a, **b**, and **c**, Force-displacement curves normalized per yarn of plain-, twill-, and satin-weave MR fabrics at different magnetic field strengths, respectively. **d**, The energy dissipation extracted from the hysteresis loop areas in **a**, **b**, and **c** as compared with single MR yarn. The shaded areas and error bars correspond to s.d. (n=3).

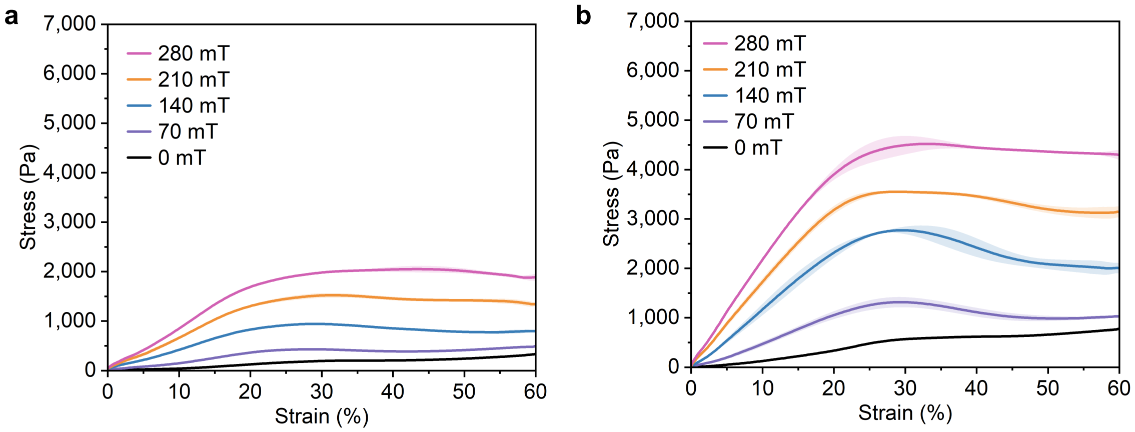

Supplementary Fig. 14. Stiffening actuation of cut-pile MR fabrics. Compression stress−strain curves with a variation of magnetic field strength of cut-pile MR fabrics with MR yarn densities of **a,** 250 and **b,** 500 yarns cm^{-2}, respectively. The shaded areas correspond to s.d. (n=3).

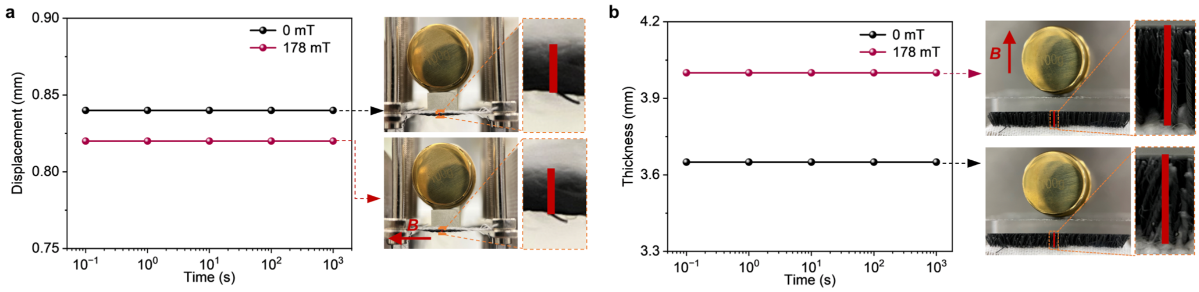

Supplementary Fig. 15. Creep property of MR fabrics. a, Creep displacement-time curves of plain weave MR fabric with horizontal magnetic field strength of 0 and 178 mT. Without applying load, the displacement is 0. **b**, Creep thickness-time curves of cut-pile MR fabric with vertical magnetic field strength of 0 and 178 mT. Thickness is 4 mm without applying pressure. The vertical red lines represent the measured displacement or thickness. Scale bars, 10 mm.

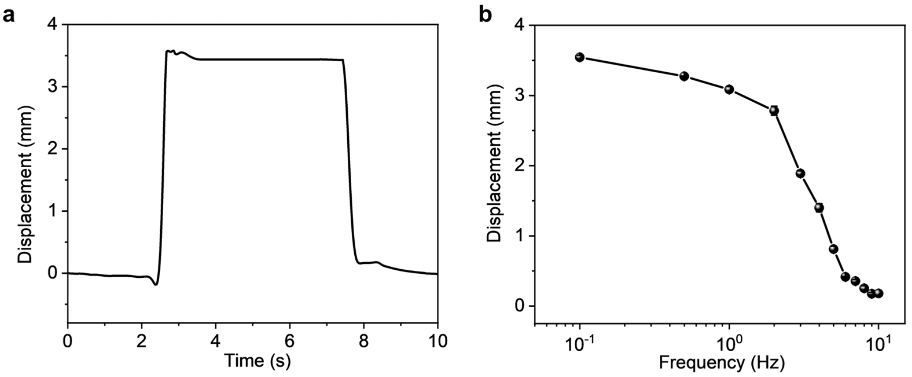

Supplementary Fig. 16. Dynamic actuation properties of the ventilation fabric. a, Displacement with time of the ventilation fabric under square wave actuation with a peak magnetic field strength of 250 mT. The ventilation fabric can quickly response to the activation magnetic field in 0.17 s, and settles at 1.2 s during this period the elastic fabric is stretched to deform. **b**, Displacement of the active ventilation fabric under square wave actuation at specific frequencies with a peak magnetic field strength of 250 mT. The actuation displacement decreases slightly from 3.5 to 2.8 mm as the frequency increases from 0.1 to 2 Hz. As the frequency continues to rise, the displacement rapidly drops to approximately 0.5 mm at 6 Hz. Error bars correspond to s.d. (n=3).

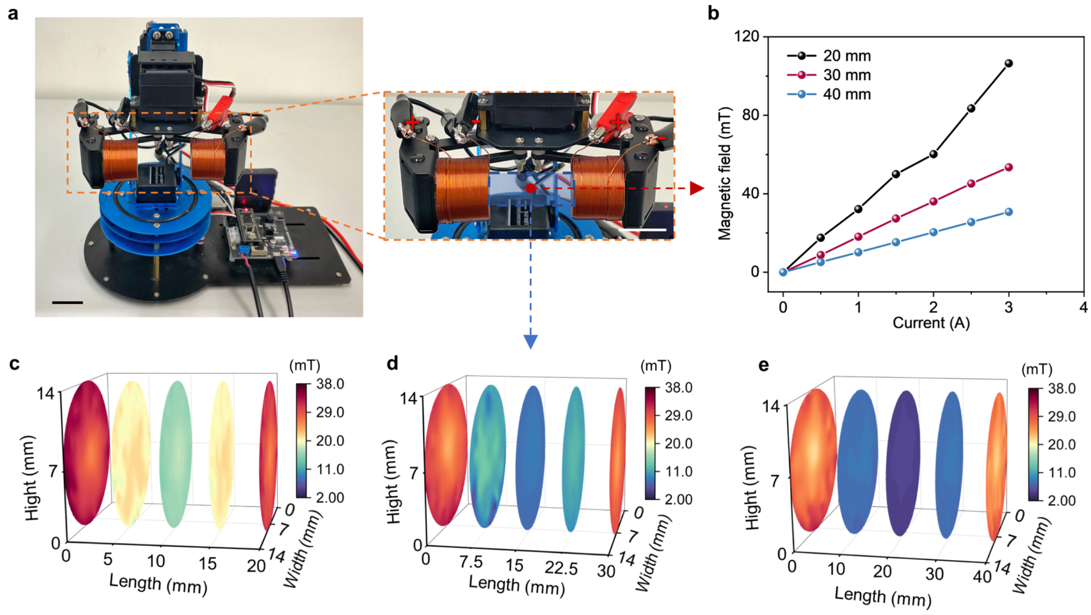

Supplementary Fig. 17. Characteristics of a mobile magnetic actuation system. a, Photograph of the mobile magnetic actuation system, consisting of two identical cylindrical electromagnets with a height of 22 mm and diameter of 29 mm. Each electromagnet has an iron core with a height of 22 mm and a diameter of 14 mm winding by 750 turns of wire with a resistance of 6.1 Ω. The electromagnets are mounted coaxially on the parallel gripper of the 6-DOF robotic arm, allowing adjustable gaps between 0 and 40 mm. The enlarged view shows the coaxially aligned electromagnets on the parallel gripper. The wires of the two electromagnets are connected in series, with indicated positive and negative poles to produce a unidirectional magnetic field when current flows through the wires. Scale bars, 2 cm. **b**, Magnetic field strength at the center between the poles for gap distances of 20, 30, and 40 mm as a function of varying electric current. **c-e**, Magnetic field strength distribution between the poles at separations of **c**, 20 mm; **d**, 30 mm; and **e**, 40 mm at a current of 0.5 A.

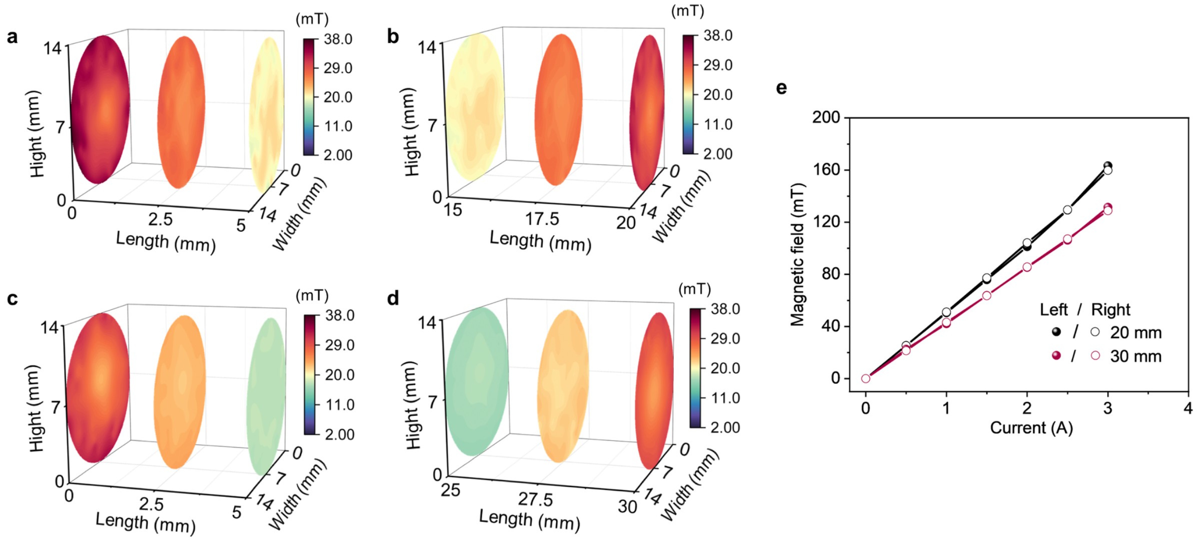

Supplementary Fig. 18. Magnetic fields for controlling cut-pile MR fabrics. a and **b**, Magnetic field strength distribution within 5 mm distance from the **a**, left and **b**, right pole surfaces at pole distance of 20 mm. The position of left and right pole is at length of 0 and 20 mm, respectively. **c** and **d**, Magnetic field strength distribution within 5 mm distance from the **c**, left and **d**, right pole surfaces at pole distance of 30 mm. The position of left and right pole is at length of 0 and 30 mm, respectively. **e**, Magnetic field strength at 2.5 mm above the center of the left and right pole surfaces for gap distances of 20 and 30 mm as a function of varying electric current.

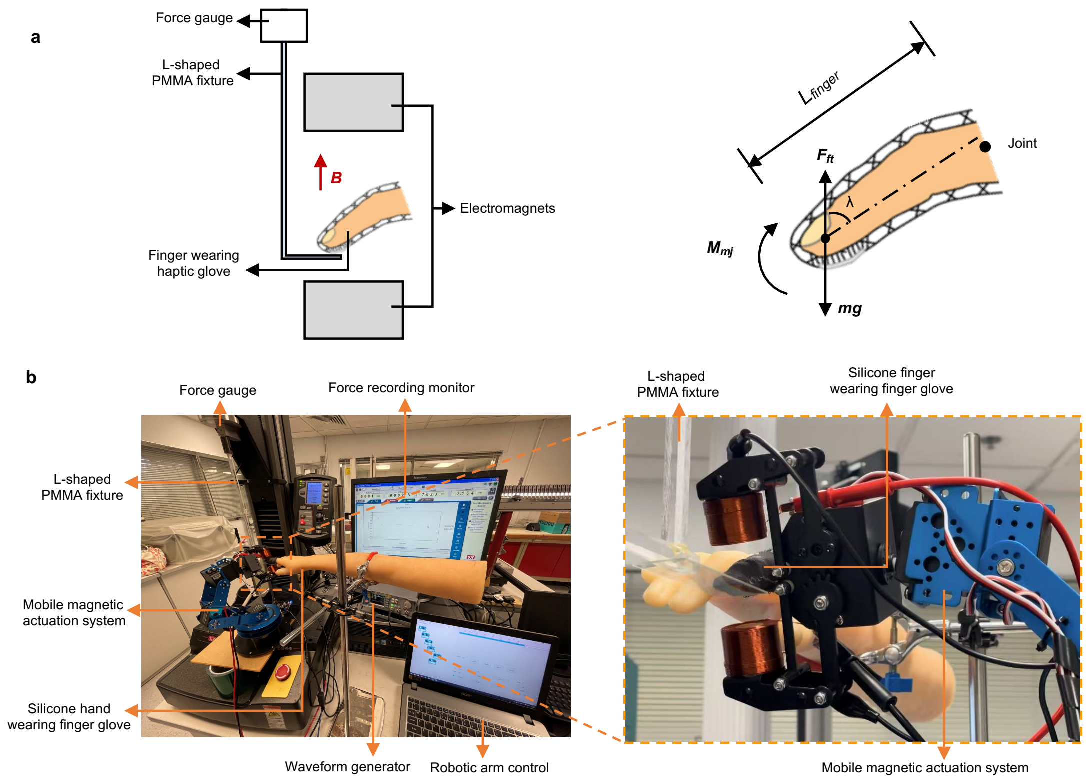

Supplementary Fig. 19. Kinesthetic feedback test. a, Schematic showing moment measurement setup and the force analysis of the finger wearing MR finger glove within magnetic field. An L-shaped PMMA fixture at the fingertip blocks the finger, allowing measurement of the relative force reduction (ΔF_{ft}) before and after magnetic field application; the resulting moment from this reduction represents the magnetic moment (M_{mj}). Misalignment between the magnetic field direction and the finger axis, representing by the angle λ, generates a moment on the MR woven fabric of the finger glove. L_{finger} and m are the length and weight of the finger, respectively. **b**, Photograph of the test setup, with a close-up view showing the magnetic actuation and force measurement section.

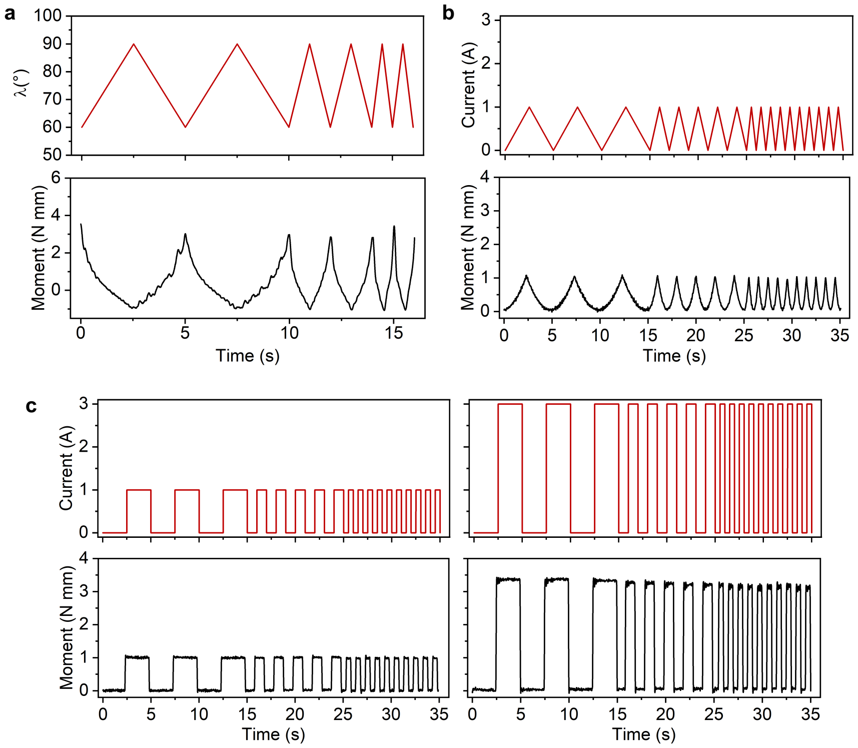

Supplementary Fig. 20. Kinesthetic effects. **a**, Input variation of angle λ between 60° and 90° at frequencies of 0.2, 0.5, and 1 Hz with a 3 A current applied to the coaxial electromagnet pair, and the resulting output moment of the MR finger glove. **b**, At λ = 60°, input current with a triangular waveform at peak currents of 1 A, and frequencies of 0.2, 0.5, and 1 Hz for the coaxial electromagnet pair, and the resulting output moment of the MR finger glove. **c**, At λ = 60°, input current with a square waveform at peak currents of 1 and 3 A, and frequencies of 0.2, 0.5, and 1 Hz for the coaxial electromagnet pair, and the resulting output moment of the MR finger glove.

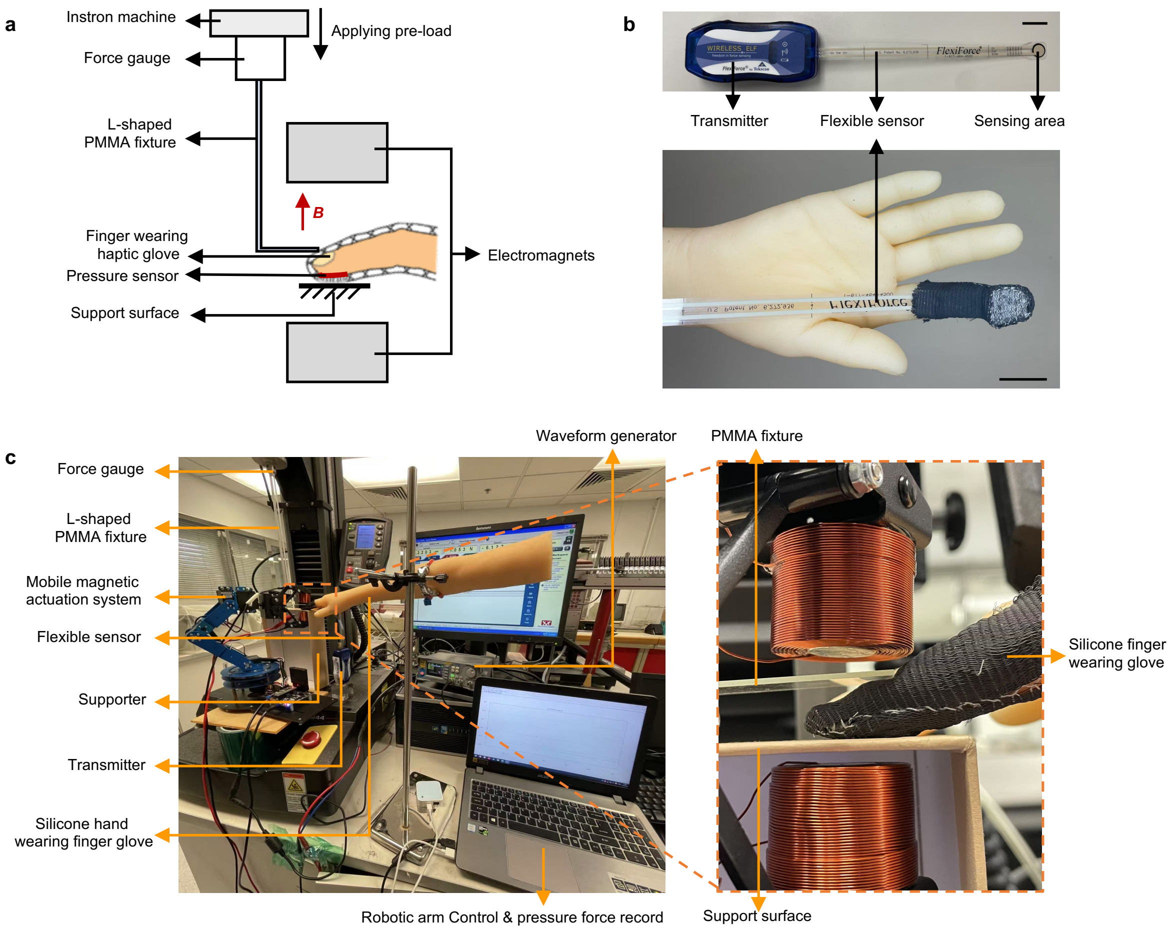

Supplementary Fig. 21. Tactile feedback test. **a**, Schematic showing pressure measurement setup. An L-shaped PMMA fixture pushes the silicone fingertip wearing MR finger glove on a flat surface to mimic the touching process. A flexible sensor is integrated between the fingertip pad and the cut-pile MR fabric to record the normal force change caused by magnetic activation. **b**, Photographs of the flexible pressure sensor with a wireless transmitter and the integration of the pressure sensor into the silicone finger wearing MR finger glove. Scale bars, 2 cm. **c**, Photograph of the test setup, with a close-up view showing the magnetic actuation and force measurement section.

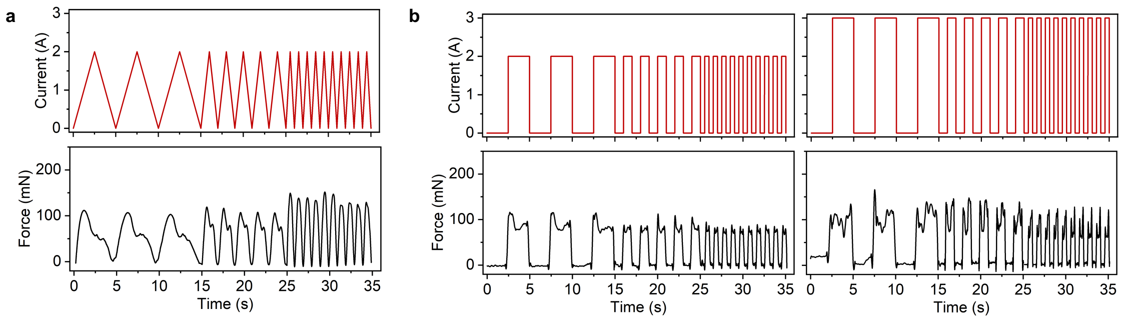

Supplementary Fig. 22. Tactile effects. **a**, At $\lambda = 90°$, with the axis of the electromagnet pair aligned across the fingertip pad, input current with a triangular waveform at peak current of 2 A, and frequencies of 0.2, 0.5, and 1 Hz for the coaxial electromagnet pair, and the resulting output force of the MR finger glove. **b**, At $\lambda = 90°$, with the axis of the electromagnet pair aligned across the fingertip pad, input current with a square waveform at peak currents of 2 and 3 A, and frequencies of 0.2, 0.5, and 1 Hz for the coaxial electromagnet pair, and the resulting output force of the MR finger glove.

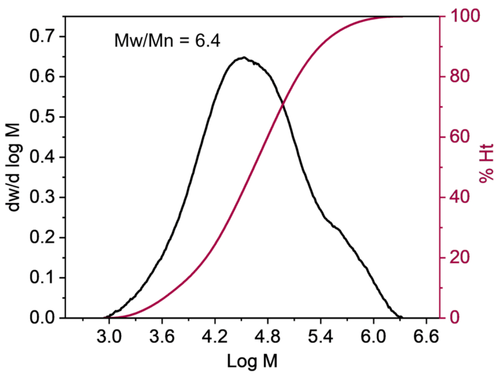

Supplementary Fig. 23. Molecular weight distribution of LDPE. The polydispersity index of LDPE is 6.4, indicating a broad molecular weight distribution.

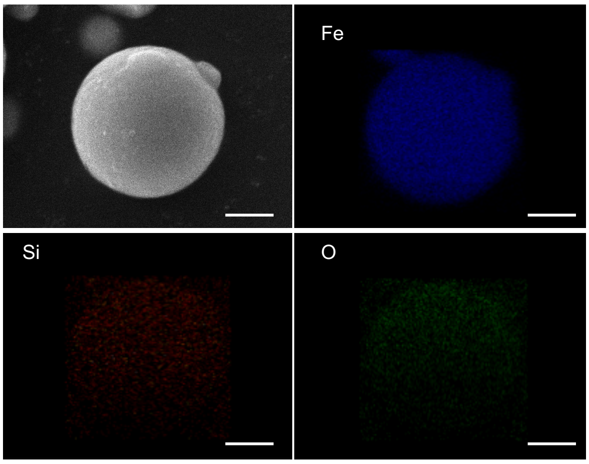

Supplementary Fig. 24. SEM and corresponding EDS elemental mapping of the CIP surface. Scale bars, 1 μm.

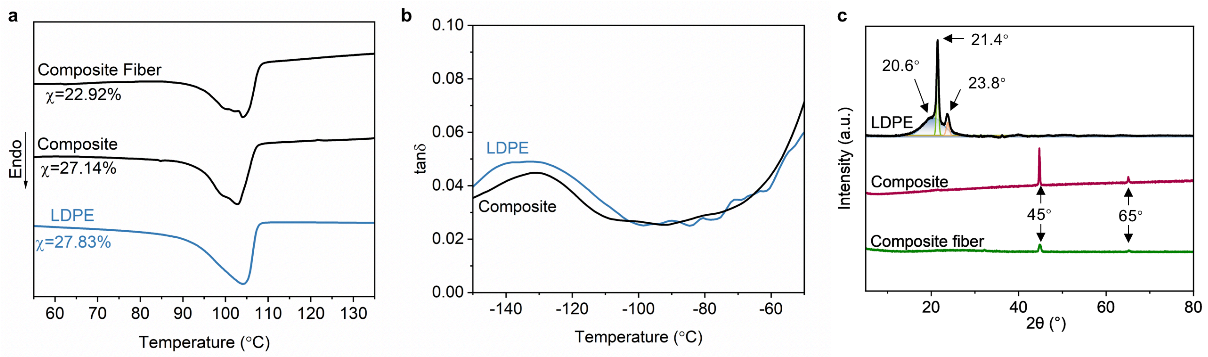

Supplementary Fig. 25. DSC, DMA and XRD of LDPE and composite. a, DSC curves of LDPE, composite with 70 wt% CIPs, and composite fiber. **b**, tan δ curves of LDPE and composite with 70 wt% CIPs. **c**, XRD patterns of LDPE, composite with 70 wt% CIPs, and composite fiber.

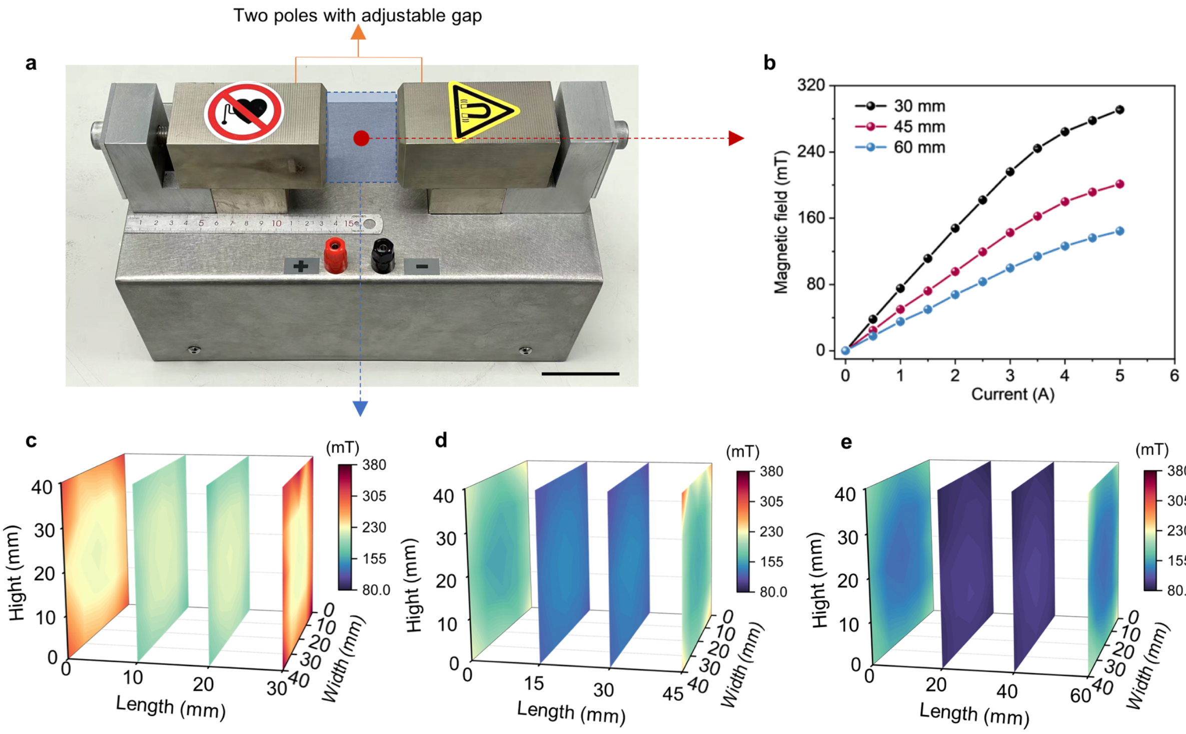

Supplementary Fig. 26. Characteristics of a stationary electromagnet. **a**, Photograph of the desktop electromagnet. Scale bar, 5 cm. **b**, Magnetic field strength at the center between the poles with gap distances of 30, 45, and 60 mm, shown as a function of varying electric current. **c-e**, Magnetic field strength distribution between the two poles at separations of **c**, 30 mm; **d**, 45 mm; and **e**, 60 mm at a current intensity of 3 A.

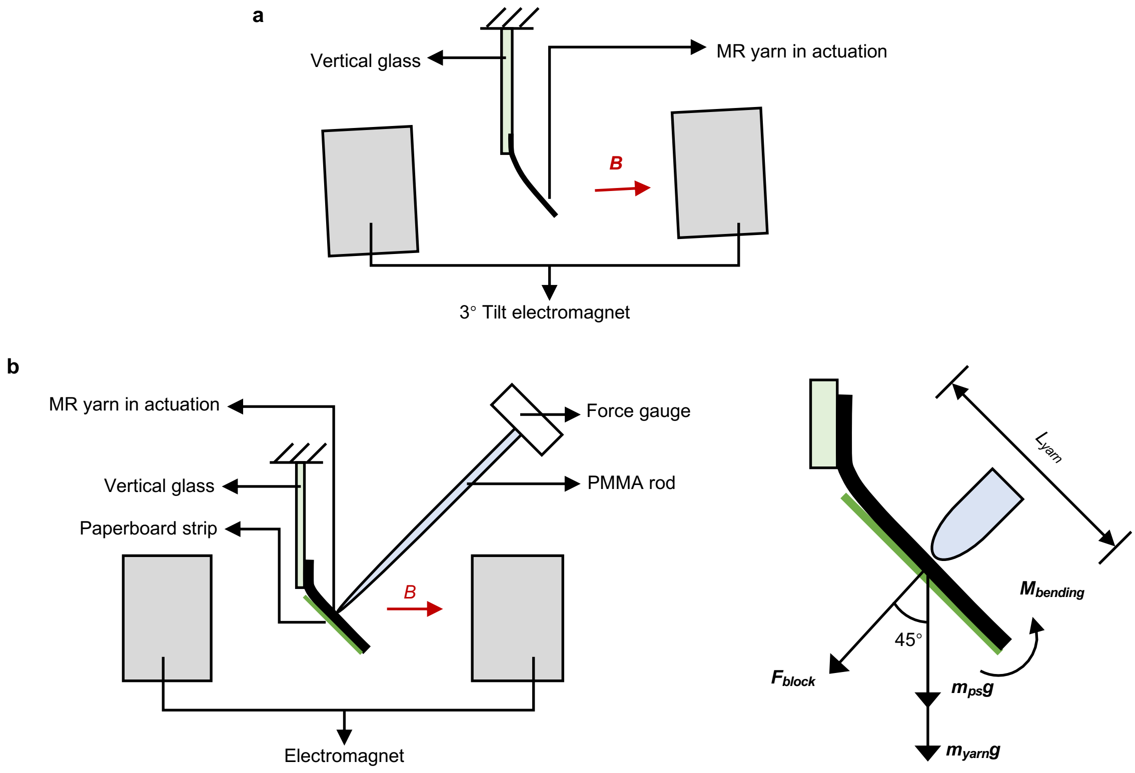

Supplementary Fig. 27. Diagrams of bending actuation test. **a**, Schematic of the setup for measuring the bending angle of MR yarn under magnetic bending actuation. **b**, Schematic of the

setup for measuring the magnetic moment density of MR yarn, including a force analysis during testing. L_{yarn} and m_{yarn} are the length and weight of the yarn, respectively. F_{block} is measured by the force gauge, m_{ps} is the weight of paperboard strip, and $M_{bending}$ is the magnetic bending moment.

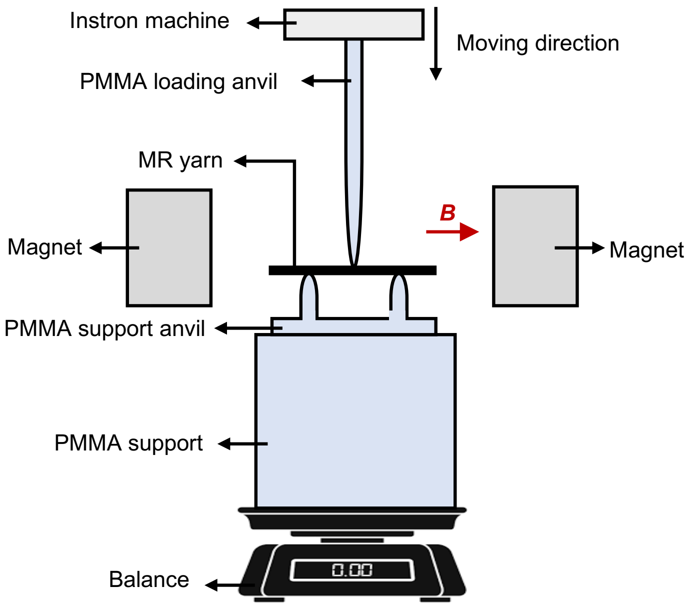

Supplementary Fig. 28. Schematic of the 3-point bending setup used to measure the magnetic stiffening effect of MR yarns.

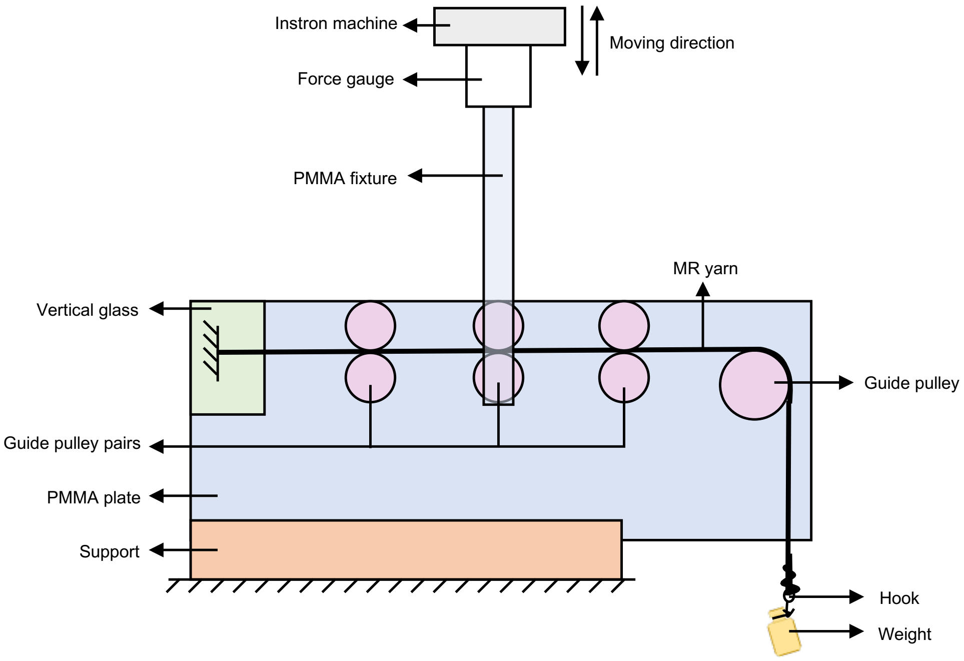

Supplementary Fig. 29. Schematic of the setup used to measure the bending durability of MR yarn.

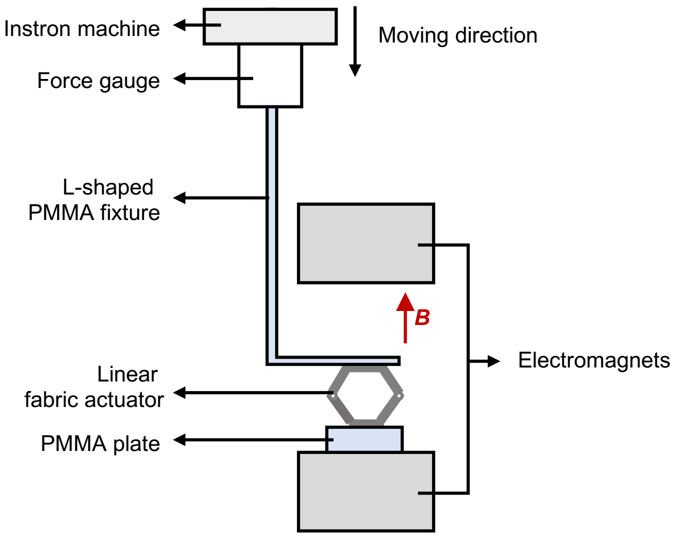

Supplementary Fig. 30. Schematic of the setup used to measure the force-stroke relationship of the fabric linear actuator under magnetic actuation.

Supplementary Table 1. Performance comparison of our SM yarn with yarns and fibers with bending or stiffening properties.

Performance / Materials (Stimulus intensity)	Safety (Time limit^)	Bending angle$^{<}$ (° mm^{-1})	Moment density* (N m kg^{-1})	Maximum flexibility (m N^{-1})	Stiffening Window (Times)	Ref.
MR yarn (300 mT)	Yes	8.29	6.5	103	29.4	This work
SMP yarns (83 °C)	No (1.5 s)	3.52	0.0015			21
PCM fiber (80 °C)	No (2 s)				40	22
SMP fiber (80 °C)	No (2 s)	4.5				23
Hydrogel fiber (80 °C)	No (2 s)			0.001$^{\#}$	30	24
SMA-SMP fiber (120 °C)	No	0.23				25
LCE-SMP fiber (90 °C)	No (0.5 s)	5.25			> 100	26
SMP-HM fiber (80 °C - 80 mT)	No (2 s)	1.15		0.02	66	27
SMP-HM fiber (80 °C - 31 mT)	No (60 s)	0.77		0.0008	71	28
HM fiber (80 mT)	Yes	5.5				29
HM fiber (unknown)		2.5	0.69			30
HM fiber (45 mT)	Yes	2.7				31
HM fiber (120 mT)	Yes	1.8				32
HM fiber (30 mT)	Yes	1.5				33
HM fiber (140 mT)	Yes		330	22$^{\#}$		34
MR fibers (20 mT)	Yes			0.03	1.21	35
DE fibers (0.8 kV)	Yes	0.12	0.0049	0.0275$^{\#}$		36
DE fibers (7 kV)	Yes	0.29	0.18			37
DE fibers (10 kV)	Yes	0.11				38
Tendon-driven fiber	Yes	11.7	0.39			39
Hydrogel fiber (water)	Yes	14				40

^ Time limit for temperature-responsive fibers and yarns to ensure safe contact with human skin, preventing burn injury.
< Bending angle is normalized by the ratio of the deflection angle of the activated bending structure to its length.
* Moment density of other work is estimated by balancing the actuation moment against gravitational moment, assuming uniform weight distribution along the length.
# Maximum flexibility is calculated by treating the material as uniform with a consistent cross-section over a 15 mm length.
Blank entries indicate properties that are either unreported or cannot be estimated from available data.

Supplementary Table 2. Several typical soft magnetic materials and their static magnetic properties at room temperature.

Type	Material	Magnetic property			Ref.
		B_s (T)	H_c (A/m)	μ_{max} (10^3)	
High-purity Fe	Fe (99.95%)	2.15	4	230	41
	Carbonyl Fe	2.15	6	20	41
Fe-Co-V alloy	$Fe_{49}Co_{49}V_2$	2.35	50	2	42
Fe-Si alloy	Oriented $Fe_{97}Si_3$	2.02	4 - 15	15 - 20	43
	Non-oriented $Fe_{96–99}Si_{1–4}$	1.96 - 2.12	30 - 80	3 - 10	43
Fe-Ni alloy	$Fe_{52}Ni_{48}$	1.6	4	100	43
	$Fe_{15}Ni_{80}Mo_5$	0.8	0.3 - 2	500	43
Amorphous/ Nanocrystalline alloy	$Fe_{78}B_{13}Si_9$	1.56	2 - 5	100	43
	$Fe_{73.5}Si_{15.5}B_7Cu_1Nb_3$	1.24	0.5	500	43
Ferrite	Sintered Mn-Zn ferrites	0.4 - 0.55	5 - 20	1 - 10	44
	Sintered Ni-Zn ferrites	0.2 - 0.35	20 - 200	0.1 - 1	45

B_s is saturation polarization, H_c is coercivity, and μ_{max} is maximum permeability.

References

1 Bertotti, G. in *Hysteresis in Magnetism* (ed Giorgio Bertotti) 103-125 (Academic Press, 1998).

2 Cēbers, A. & Javaitis, I. Bending of flexible magnetic rods. *Physical Review E* **70**, 021404, doi:10.1103/PhysRevE.70.021404 (2004).

3 Benjamin, E. & Rich, S. in *Biomimetic Based Applications* (ed George Anne) Ch. 17 (IntechOpen, 2011).

4 Erb, R. M., Martin, J. J., Soheilian, R., Pan, C. & Barber, J. R. Actuating Soft Matter with Magnetic Torque. *Advanced Functional Materials* **26**, 3859-3880, doi:https://doi.org/10.1002/adfm.201504699 (2016).

5 Kim, Y. & Zhao, X. Magnetic Soft Materials and Robots. *Chem Rev* **122**, 5317-5364, doi:10.1021/acs.chemrev.1c00481 (2022).

6 Costello, G. A. in *Theory of Wire Rope* (ed George A. Costello) 11-43 (Springer New York, 1997).

7 Mrlik, M. *et al.* Cholesteryl-coated carbonyl iron particles with improved anti-corrosion stability and their viscoelastic behaviour under magnetic field. *Colloid and Polymer Science* **292**, 2137-2143, doi:10.1007/s00396-014-3245-5 (2014).

8 Strååt, M., Toll, S., Boldizar, A., Rigdahl, M. & Hagström, B. Melt spinning of conducting polymeric composites containing carbonaceous fillers. *Journal of Applied Polymer Science* **119**, 3264-3272, doi:https://doi.org/10.1002/app.32882 (2011).

9 Liu, C., He, J., Ruymbeke, E. v., Keunings, R. & Bailly, C. Evaluation of different methods for the determination of the plateau modulus and the entanglement molecular weight. *Polymer* **47**, 4461-4479, doi:https://doi.org/10.1016/j.polymer.2006.04.054 (2006).

10 Pourazadi, S., Shagerdmootaab, A., Chan, H., Moallem, M. & Menon, C. On the electrical safety of dielectric elastomer actuators in proximity to the human body. *Smart Materials and Structures* **26**, 115007, doi:10.1088/1361-665X/aa89b1 (2017).

11 He, X., Ye, A., Fu, X., Yang, W. & Wang, Y. Achieving Low-Energy-Barrier Ion Hopping in Adhesive Composite Polymer Electrolytes by Nanoabsorption. *Macromolecules* **55**, 7117-7126, doi:10.1021/acs.macromol.2c00928 (2022).

12 Kubát, J., Rigdahl, M. & Welander, M. Characterization of interfacial interactions in high density polyethylene filled with glass spheres using dynamic-mechanical analysis. *Journal of Applied Polymer Science* **39**, 1527-1539, doi:https://doi.org/10.1002/app.1990.070390711 (1990).

13 Wu, T., Li, Y. & Wu, G. Crystalline structure and phase structure of mLLDPE/LDPE blends. *Polymer* **46**, 3472-3480, doi:https://doi.org/10.1016/j.polymer.2005.02.084 (2005).

14 Lafrance, C. P., Pezolet, M. & Prud'homme, R. E. Study of the distribution of molecular orientation in highly oriented polyethylene by x-ray diffraction. *Macromolecules* **24**, 4948-4956, doi:10.1021/ma00017a035 (1991).

15 Liu, L., Duan, Y., Liu, S., Chen, L. & Guo, J. Microwave absorption properties of one thin sheet employing carbonyl–iron powder and chlorinated polyethylene. *Journal of Magnetism and Magnetic Materials* **322**, 1736-1740, doi:https://doi.org/10.1016/j.jmmm.2009.12.017 (2010).

16 Mahmoud, M. E. *et al.* Fabrication, characterization and gamma rays shielding properties of nano and micro lead oxide-dispersed-high density polyethylene composites. *Radiation Physics and Chemistry* **145**, 160-173, doi:https://doi.org/10.1016/j.radphyschem.2017.10.017 (2018).

17 Feng, C.-P. *et al.* A Facile Route to Fabricate Highly Anisotropic Thermally Conductive Elastomeric POE/NG Composites for Thermal Management. *Advanced Materials Interfaces* **5**, 1700946, doi:https://doi.org/10.1002/admi.201700946 (2018).

18 McNally, T. *et al.* Polyethylene multiwalled carbon nanotube composites. *Polymer* **46**, 8222-8232, doi:https://doi.org/10.1016/j.polymer.2005.06.094 (2005).

19 Feng, C.-P. *et al.* Robust polymer-based paper-like thermal interface materials with a through-plane thermal conductivity over 9 Wm−1K−1. *Chemical Engineering Journal* **392**, 123784, doi:https://doi.org/10.1016/j.cej.2019.123784 (2020).

20 Feng, C.-P. *et al.* Multifunctional Thermal Management Materials with Excellent Heat Dissipation and Generation Capability for Future Electronics. *ACS Applied Materials & Interfaces* **11**, 18739-18745, doi:10.1021/acsami.9b03885 (2019).

21 Madani, Z. *et al.* Light-Driven Multidirectional Bending in Artificial Muscles. *Advanced Materials* **36**, 2405917, doi:https://doi.org/10.1002/adma.202405917 (2024).

22 Wang, R.-q. *et al.* Weavable phase change fibers with wide thermal management temperature range, reversible thermochromic and triple shape memory functions towards human thermal management. *European Polymer Journal* **187**, 111890, doi:https://doi.org/10.1016/j.eurpolymj.2023.111890 (2023).

23 Strutynski, C. *et al.* 4D Optical fibers based on shape-memory polymers. *Nature Communications* **14**, 6561, doi:10.1038/s41467-023-42355-7 (2023).

24 Wu, J., Wu, B., Xiong, J., Sun, S. & Wu, P. Entropy-Mediated Polymer–Cluster Interactions Enable Dramatic Thermal Stiffening Hydrogels for Mechanoadaptive Smart Fabrics. *Angewandte Chemie International Edition* **61**, e202204960, doi:https://doi.org/10.1002/anie.202204960 (2022).

25 Du, H., Yao, Y., Liu, Y. & Zhao, W. Two-Way Shape Memory Effect of a Shape Memory Composite Strip. *Applied Sciences* **13** (2023).

26 Li, M. *et al.* Coaxial-Spun Hollow Liquid Crystal Elastomer Fiber as a Versatile Platform for Functional Composites. *Advanced Functional Materials* **34**, 2406847, doi:https://doi.org/10.1002/adfm.202406847 (2024).

27 Piskarev, Y. *et al.* Fast-Response Variable-Stiffness Magnetic Catheters for Minimally Invasive Surgery. *Advanced Science* **11**, 2305537, doi:https://doi.org/10.1002/advs.202305537 (2024).

28 Richter, M., Venkiteswaran, V. K. & Misra, S. Concentric Tube-Inspired Magnetic Reconfiguration of Variable Stiffness Catheters for Needle Guidance. *IEEE Robotics and Automation Letters* **8**, 6555-6562, doi:10.1109/LRA.2023.3307294 (2023).

29 Kim, Y. *et al.* Telerobotic neurovascular interventions with magnetic manipulation. *Science Robotics* **7**, eabg9907, doi:10.1126/scirobotics.abg9907.

30 Fan, J. *et al.* Magnetic Fiber Robots with Multiscale Functional Structures at the Distal End. *Advanced Functional Materials* **34**, 2309424, doi:https://doi.org/10.1002/adfm.202309424 (2024).

31 Zhang, Y. *et al.* Submillimeter Multifunctional Ferromagnetic Fiber Robots for Navigation, Sensing, and Modulation. *Advanced Healthcare Materials* **12**, 2300964, doi:https://doi.org/10.1002/adhm.202300964 (2023).

32 Zhang, Y. *et al.* Coaxially printed magnetic mechanical electrical hybrid structures with actuation and sensing functionalities. *Nature Communications* **14**, 4428, doi:10.1038/s41467-023-40109-z (2023).

33 Mao, L. *et al.* Magnetic steering continuum robot for transluminal procedures with programmable shape and functionalities. *Nature Communications* **15**, 3759, doi:10.1038/s41467-024-48058-x (2024).

34 Banerjee, H. *et al.* Soft Multimaterial Magnetic Fibers and Textiles. *Advanced Materials* **35**, 2212202, doi:https://doi.org/10.1002/adma.202212202 (2023).

35 Atakuru, T., Kocabaş, F., Pagliarani, N., Cianchetti, M. & Samur, E. Fiber Jamming of Magnetorheological Elastomers as a Technique for the Stiffening of Soft Robots. *Robotics* **13** (2024).

36 He, J. *et al.* Intrinsically Anisotropic Dielectric Elastomer Fiber Actuators. *ACS Materials Letters* **4**, 472-479, doi:10.1021/acsmaterialslett.1c00742 (2022).

37 Chortos, A. *et al.* Printing Reconfigurable Bundles of Dielectric Elastomer Fibers. *Advanced Functional Materials* **31**, 2010643, doi:https://doi.org/10.1002/adfm.202010643 (2021).

38 Shimizu, K., Nagai, T. & Shintake, J. Dielectric Elastomer Fiber Actuators with Aqueous Electrode. *Polymers* **13** (2021).

39 Leber, A. *et al.* Highly Integrated Multi-Material Fibers for Soft Robotics. *Advanced Science* **10**, 2204016, doi:https://doi.org/10.1002/advs.202204016 (2023).

40 Zhai, Y., Gong, C., Chen, J. & Chang, C. Magnetic-field induced asymmetric hydrogel fibers for tough actuators with programmable deformation. *Chemical Engineering Journal* **477**, 147088, doi:https://doi.org/10.1016/j.cej.2023.147088 (2023).

41 Tumanski, S. *Handbook of magnetic measurements*. (CRC press, 2016).

42 He, J. *et al.* Soft magnetic materials for power inductors: State of art and future development. *Materials Today Electronics* **6**, 100066, doi:https://doi.org/10.1016/j.mtelec.2023.100066 (2023).

43 Park, B. J., Fang, F. F. & Choi, H. J. Magnetorheology: materials and application. *Soft Matter* **6**, 5246-5253, doi:10.1039/C0SM00014K (2010).

44 Talaat, A. *et al.* Review on soft magnetic metal and inorganic oxide nanocomposites for power applications. *Journal of Alloys and Compounds* **870**, 159500, doi:https://doi.org/10.1016/j.jallcom.2021.159500 (2021).

45 Carlson, J. D. & Jolly, M. R. MR fluid, foam and elastomer devices. *Mechatronics* **10**, 555-569, doi:https://doi.org/10.1016/S0957-4158(99)00064-1 (2000).
